# An integrative analysis of elementomics and oxidation-reduction potentials identifies redox-related metals associated with coronary artery disease and post-PCI outcomes in type 2 diabetic patients

**DOI:** 10.1016/j.redox.2026.104270

**Published:** 2026-06-19

**Authors:** Zi-Qi Fang, Bing Wang, Ning-Hua Cui, Zhe-Zhe Yang, Xue-Bin Wang

**Affiliations:** a Department of Clinical Laboratory, Key Clinical Laboratory of Henan Province, The First Affiliated Hospital of Zhengzhou University, Zhengzhou, Henan, 450000, China; b Department of Clinical Laboratory, Children's Hospital Affiliated to Zhengzhou University, Zhengzhou Key Laboratory of Children's Infection and Immunity, Zhengzhou, Henan, 450000, China

**Keywords:** Oxidation-reduction potential, Markers of the global redox status, Elementomics, Diabetes, Bioenergetics

## Abstract

Exposure to metals has been associated with elevated oxidative stress, which may contribute to diabetic macrovascular complications. However, current methods for evaluating oxidative stress, which mainly rely on quantifying individual byproducts of oxidative damage, may not fully characterize the effects of metal exposure on global redox imbalance, especially in the setting of type 2 diabetes mellitus (T2DM). Here, by assessing global redox status using the novel marker oxidation-reduction potential (ORP) and performing elemental profiling of 35 plasma metals/metalloids in a large cohort of 3142 patients with T2DM, we successfully identified a mixture of 5 redox-related metals (including nickel, zirconium, titanium, strontium, and vanadium), which showed strong associations not only with ORP, but with conventional markers of protein, lipid, and DNA oxidation. Importantly, high exposure to the redox-related metal mixture cross-sectionally correlated with obstructive coronary artery disease (CAD) and prospectively predicted adverse events after percutaneous coronary intervention (PCI) in patients with T2DM. Further cytokine profiling found that changes in cytokines within the nuclear factor-kappa B (NF-κB) pathway might mediate the associations of the redox-related metal mixture with obstructive CAD and post-PCI outcomes. Notably, *ex vivo* experiments of peripheral blood mononuclear cells (PBMCs) showed that exposure to redox-related metals induced a specific pattern of bioenergetic dysfunction characterized by impaired respiration of mitochondrial complexes I and III, leading to mitochondrial oxidant overproduction and consequent NF-κB activation. Collectively, our findings indicate ORP as a promising marker of metal-induced oxidative stress and support the links of redox-related metals to cardiometabolic risk in patients with T2DM.

## Highlights

• Integration of ORP and elementomics identifies redox-related metals in patients with T2DM.
• High exposure to redox-related metals increases CAD risk and predicts post-PCI outcomes.
• Cytokines within the NF-κB pathway mediate the adverse effects of redox-related metals.
• Redox-related metals induce a specific patterns of bioenergetic dysfunction in PBMCs.

## Graphical abstract

**Figure:**
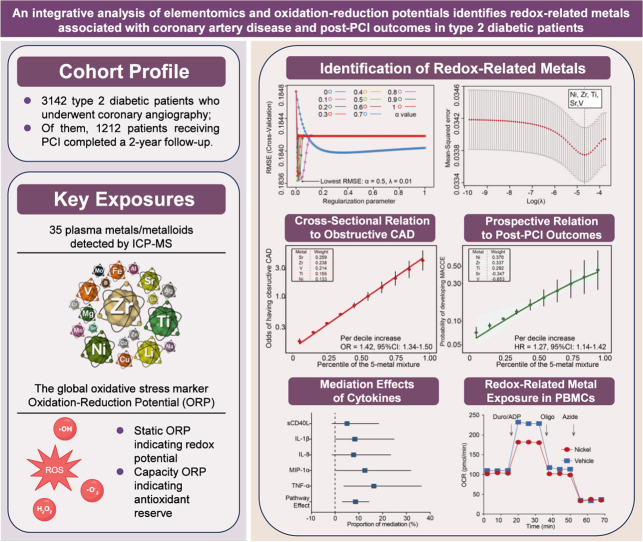

## Abbreviations

T2DM: indicates type 2 diabetes mellitus
CAD: coronary artery disease
PCI: percutaneous coronary intervention
ROS: reactive oxygen species
ORP: oxidation-reduction potential
PBMC: peripheral blood mononuclear cell
MACCE: major adverse cardiovascular and cerebrovascular events
MDA: malondialdehyde
8-OHdG: 8-hydroxy-2′-deoxyguanosine, 8-OHdG
NF-κB: nuclear factor-kappa B

## 1 Introduction

Patients with type 2 diabetes mellitus (T2DM) are at high risk of coronary artery disease (CAD) and cardiovascular death [1,2]. Although percutaneous coronary intervention (PCI) has been evolved to provide more effective treatment for obstructive CAD, adverse ischaemic events after PCI are still frequent and significantly burdened by poor outcomes in patients with T2DM [3]. These may, in part, be explained by the induction of oxidative stress under hyperglycemic milieus [4], which provides conditions for systemic inflammation and vascular damage [5].

Circulating metals and metalloids arising from both anthropogenic and natural sources have been proposed as a class of inducers of oxidative stress, with direct effects on overproducing reactive oxygen species (ROS) [6] and indirect effects on deleting the antioxidant glutathione [7]. Recently, emerging evidence has demonstrated that contaminant metals play essential roles as modifiable risk factors for cardiovascular disease in the general population [8,9]. However, there is limited information on the associations of metal profiles with CAD risk and post-PCI outcomes in patients with T2DM. Importantly, although previous reports have correlated metal exposure with altered levels of individual byproducts of oxidative damage [10,11], very little is known about the effects of metal mixture on the overall redox status and the links of metal-mediated redox imbalance to cardiovascular outcomes and inflammatory status, especially in the setting of T2DM.

Here, in a large cohort of 3142 type 2 diabetic patients, we first performed an elementomics approach to characterize a total of 35 plasma metals/metalloids, and then used a novel technique to measure plasma oxidation-reduction potentials (ORPs) for global evaluation of redox potential and antioxidant reserve [12]. By integrating elemental profiles and ORP values, we aimed to 1) identify a panel of metal mixture with significant impacts on the overall redox status, 2) assess whether the redox-related metal mixture cross-sectionally correlated with obstructive CAD and concentrations of inflammatory cytokines and prospectively predicted adverse events after PCI, and 3) provide *ex vivo* evidence for the bioenergetic effects of the redox-related metals on the redox, respiratory, and inflammatory states of peripheral blood mononuclear cells (PBMCs). Our study has important implications for comprehensive characterization of redox-related metals and for the use of ORP as a marker of metal-mediated oxidative stress and cardiovascular risk in the context of T2DM.

## 2 Methods

### 2.1 Materials

Information on reagents and commercial kits used in this study is summarized in Supplementary Table 1.

### 2.2 Study population

Detailed information on study design, baseline characteristics, and outcome definitions has been summarized in Supplementary Methods [[13], [14], [15], [16]]. Briefly, we initially recruited a total of 3642 type 2 diabetic patients who underwent coronary angiography due to angina or angina equivalent symptoms between Jun 2016 and Nov 2019 at 2 cardiac centers in China (the First Affiliated Hospital of Zhengzhou University and the Zhongnan Hospital of Wuhan University). At baseline, demographic, lifestyle, and clinical data were collected through face-to-face interviews with patients and medical history examinations; venous blood samples were drawn before angiography. Patients were excluded if they 1) were aged <40 years (n = 59), 2) were unable to donate sufficient blood samples (n = 57), 3) had a history of cancer, abnormal liver or renal function, and serious infection (n = 240), and 4) had a history of other cardiac diseases (e.g., valvular disease, congenital heart disease, and rheumatic heart disease, n = 144). Finally, we obtained full data from 3142 patients with T2DM for cross-sectional analyses. To compare the elemental profiles between type 2 diabetic patients and nondiabetic subjects, we also included a total of 1000 subjects without T2DM confirmed by physical examinations.

In this study, T2DM was diagnosed according to the ADA 2014 criteria [17]. Indications for angiography were defined based on the ACC/AHA 2012 guideline [18]. Obstructive CAD was angiographically confirmed as stenosis of >50% in at least 1 major coronary artery. The severity of coronary stenosis was evaluated using an angiographic CAD index, which created 7 categories of CAD severity: no apparent CAD (<20% stenosis); 1-, 2-, and 3-vessel nonobstructive CAD (20%-50% stenosis); 1-, 2-, and 3-vessel obstructive CAD (>50% stenosis), based on the extent of maximal stenosis and the number of diseased vessels [[13], [14], [15]]. As recommended by Maddox et al. [19], patients with isolated 20%-49% left main coronary stenosis were classified into the 1-vessel nonobstructive CAD group; patients with >50% left main stenosis were included into the 3-vessel obstructive CAD group.

Of the 3142 type 2 diabetic patients, 1212 who received primary PCI within 30 days after angiography completed a 2-year follow-up survey for prospectively tracking post-PCI outcomes. The primary outcome was major adverse cardiovascular and cerebrovascular events (MACCE), a composite of all-cause death, non-fatal myocardial infarction, non-fatal stroke, and repeat revascularization [20]. The secondary outcomes comprised individual components of MACCE and cardiovascular death [16]. Outcome data were documented through telephone interviews with patients and medical record reviews at 30 days, 1 year, and 2 years after PCI, and centrally adjudicated by 2 cardiologists who were blinded to the baseline data. For all patients undergoing PCI, the SYNTAX II score was calculated at baseline for risk prediction of post-PCI outcomes [21]. The study protocols were approved by local Institutional Review Boards. All participants provided written informed consents.

### 2.3 Assessment of the overall redox status by plasma ORP

Plasma ORP values were measured using the MioxSYS® System (Aytu BioPharma, Denver, USA) following the manufacturer's instructions. Briefly, plasma samples were freshly isolated, pipetted onto a MioxSYS® sensor containing 3 electrodes (working, counter, and reference), and subsequently inserted into a MioxSYS® analyzer. After applying a low oxidizing current (1 nA), the analyzer measured the potential difference between the working and reference electrodes, which provided a static ORP (sORP, mV) reading for evaluation of the balance between total oxidants and reductants, thus reflecting the overall state of the oxidative stress in a plasma sample [22]. Then, a linearly increasing oxidizing current was applied until the charge between the counter and working electrodes changed at the maximum rate, which indicated that all readily oxidizable molecules were oxidized [22]. Thus, the capacity ORP (cORP, μCoulomb) values could be measured to represent the antioxidant reserve of a plasma sample [12].

To test the reliability of the ORP measurement, ascorbic acid solutions with test concentrations of 10 μM, 20 μM, 50 μM, and 100 μM were used to generate a standard curve relating to the measured potential [12]. Using this standard curve to calculate the concentration of a 40 μM ascorbic acid solution showed a result of 39.9 ± 1.5 μM with an error of 1.2% ± 0.5%, confirming the accuracy of the ORP measurement. Then, 3 control samples, prepared by pooling plasma samples with different ORP values, were used to estimate the between-run precision [23], which was within an acceptable range for both sORP (CVs: 5.4%-10.5%) and cORP (CVs: 5.5%-9.4%). Finally, to test whether ORP readings reflect true redox potential, a pooled plasma sample was titrated by adding increasing concentrations of 0.1%, 0.3%, 0.5%, and 0.7% of H_2_O_2_ solutions. Triplicate measurements of ORP in 3 different days showed that addition of increasing concentrations of exogenous H_2_O_2_ led to a linear increase in sORP signals and a linear decrease in cORP signals (Supplementary Fig. 1), indicating that the ORP measurement is sensitive to perturbation by a potent oxidant agent [24].

### 2.4 Quantification of conventional redox markers in plasma

Protein carbonyl content, as a marker of protein oxidation [25], was measured in plasma based on the reaction of DNPH with protein carbonyls, in which DNP hydrazones were formed and colorimetrically quantifiable at 375 nm. To correct for variability in protein abundance, the measurements were standardized by total protein levels in plasma (measured by the Bradford reagent) and expressed as nmol/mg protein. Malondialdehyde (MDA, μM), as the major product of lipid peroxidation [26], was quantified by colorimetrically monitoring the formation of the MDA-thiobarbituric acid adduct at 532 nm. 8-hydroxy-2′-deoxyguanosine (8-OHdG, ng/mL), as a marker of oxidative DNA damage [27], was determined using the Abcam ELISA kit, following the manufacturer's instructions. Metallothionein (MT, pg/mL), as a protective protein against metal-induced oxidative stress [28], was detected using the ELISA assay (Cusabio). The reduced (GSH) and oxidized (GSSG) forms of glutathione (μM), as key components of antioxidant systems, were measured using high-performance liquid chromatography (HPLC) [29].

### 2.5 Elementomics by inductively coupled plasma-mass spectrometry

Following previously reported protocols [30], plasma elemental profiling was performed using an inductively coupled plasma-mass spectrometer with an octupole-based collision/reaction cell (Agilent 7850 ICP-MS, Santa Clara, USA). Samples were analyzed in random order to mitigate the potential batch effects. The concentrations (μg/L) of 35 elements (metals/metalloids) were estimated with the 5-point standard curves with R coefficients greater than 0.99. For each sample, the average of 3 replicate readings was reported as the element concentrations. The limit of detection (LOD) was calculated as 3 times the average of 10 consecutive measurements of the blank diluent [30]. For 30 elements with high detection rates of >90% (Supplementary Table 2), concentrations below LOD were imputed with the values of LOD/2. The remaining 5 elements (beryllium, silver, tantalum, wolfram, and rhenium) were categorized as “detectable” and “undetectable” for subsequent analyses, due to low detection rates (all <50%) [30]. For quality control (QC), 2 certified QC samples (ClinChek® human plasma controls for trace elements, No. 8883 and No. 8884) were inserted in each batch. The measured values of the QC samples were within the recommended range. The intra-assay and inter-assay CVs were less than 10% for all analyzed elements. Detailed procedures of elementomics are presented in Supplementary Methods.

### 2.6 Measurements of inflammatory cytokines and adipokines in plasma

Using previous knowledge from the KEGG database, we selected a total of 31 inflammatory proteins (cytokines and adipokines) from 5 pathways with causal effects on cardiometabolic health: 1) the Janus kinase signal transducer and activator of transcription proteins signaling pathway [31]; 2) the nuclear factor-kappa B (NF-κB) pathway [32]; 3) the chemokine signaling pathway [33]; 4) the adipocytokine signaling pathway [34]; and 5) the cholesterol metabolism pathway [35]. Plasma concentrations of the 31 selected proteins were measured with the customized Human Cytokine/Chemokine/Growth Factor 20-plex magnetic panel, the Human Adipokine 5-plex magnetic panel, and the Human Apolipoprotein 6-plex magnetic panel (MILLIPLEX) using a Luminex xMAP multiplex platform. For protein quantification, an 8-point standard curve was prepared within each plate using the serially diluted calibrators. Raw intensities obtained with the Luminex xMAP platform (Thermo Fisher Scientific, Waltham, USA) were converted to the concentration values (pg/mL) using the calculated standard curves. Plate batch effect was corrected for each concentration value by subtracting the difference between the overall protein average minus the plate-specific protein average [36]. The CVs for each protein, estimated by plate and then averaged, ranged from 4.4% to 13.8%. The minimum detectable concentration (MinDC) of each protein was calculated using MILLIPLEX® Analyst 5.1. Values below MinDC (<10% for all proteins) were imputed with the values of MinDC/2.

### 2.7 Ex vivo experiments in PBMCs

Details of the *ex vivo* experiments have been introduced in Supplementary Methods. Briefly, PBMCs of 15 participants without obstructive CAD were isolated by density gradient centrifugation, counted by a hemocytometer, and resuspended at a density of 2.5 × 10^5^/mL. For mimicking metal exposure, freshly isolated PBMCs were pretreated for 24 h with 50 ng/mL nickel chloride (NiCl_2_⋅6H_2_O), 100 ng/mL titanium dioxide nanoparticles, or 10 ng/mL zirconia nanoparticles. Cell viability and apoptosis of PBMCs were determined by MTT assay and Annexin-V/PI double staining, respectively. The activity of citrate synthase, as a strong indicator of mitochondrial content [37], was detected using the colorimetric assay (Abcam).

Bioenergetics of PBMCs were measured with a Seahorse XFe96 Analyzer (Agilent, Santa Clara, USA), in which the rates of mitochondrial respiration and glycolytic efflux were monitored in real time according to the protocols of the Mito Stress test and the Glycolytic Rate assay, respectively [38]. The respiratory activities of individual electron transport chain (ETC) complexes (complex I [CI] to complex IV [CIV]) were determined by incubating PBMCs with 25 μg/mL saponin first (for permeabilization), followed by injection of specific substrates for each complex and monitoring of state 3 and state 4 respiration by sequential addition of 1 mM ADP and 1 μg/mL oligomycin [39]. The enzymatic activities of individual ETC complexes (CI-CV) were detected using the spectrophotometric assays as introduced by Medja et al. [40].

The rate of mitochondrial superoxide production in PBMCs was measured by staining with the non-fluorescent probe dihydrorhodamine 123 (5 μM), which can irreversibly react with ROS to form the highly fluorescent rhodamine 123 in mitochondria. The increment in fluorescence of rhodamine 123 was continuously monitored by a plate reader (BMG LABTECH, Germany), and then fitted by linear regression to estimate the regression slope, which represented the average rate of mtROS production over time [38]. Considering the practical challenge of measurement of short-lived superoxide [41], the rate of mitochondrial H_2_O_2_ release was also detected by first isolating mitochondria from PBMCs using the Qiagen Mitochondria Isolation kit, followed by conversion of mitochondrial superoxide into H_2_O_2_ by the addition of superoxide dismutase and continuous measurement of the reaction of the Amplex Red system (Invitrogen) with H_2_O_2_ by a plate reader. The following 2 protocols were used to specifically model H_2_O_2_ release by CI and CIII, the 2 major sources of mtROS [42]. First, freshly isolated mitochondria were incubated first with the CI substrates pyruvate (5 mM) and malate (2.5 mM), and then with the CI inhibitor rotenone (0.5 μM) for inducing the maximal release of H_2_O_2_ at CI [42]. Second, following the addition of the CII substrate succinate (10 mM), isolated mitochondria were exposed to rotenone that blocks reverse electron flow to CI and antimycin A (0.5 μM) that induces the maximal release of H_2_O_2_ at the Qo superoxide generation site of CIII [43].

Quantification of GSH/GSSG and NADPH/NADP+ was performed using the HPLC assay [29] and the fluorometric assay (Abcam), respectively. The activity of the NF-κB subunits (p50, p52, p65, c-Rel, and RelB) was quantitatively analyzed using the TransAM DNA-Binding ELISA assay (Active Motif) according to the manufacturer's instructions [44]. The mRNA expression and protein secretion of NF-κB-dependent proinflammatory cytokines were determined by reverse transcription quantitative PCR and Luminex xMAP technology, respectively.

### 2.8 Statistical analysis

We conducted descriptive analyses by baseline characteristics using the Kruskal-Wallis test (for multi-group comparisons) and the Mann-Whitney *U* test (for 2-group comparisons). ORP and metal levels were right-skewed and therefore ln-transformed for subsequent analyses. We applied multiple linear regression models to estimate the percent changes in ORP values by each metal, with the Benjamini-Hochberg method for controlling the false discovery rate (FDR) in multiple comparisons [45]. Metal levels were 1) analyzed continuously, 2) categorized into quartiles, and 3) modeled using restricted cubic splines to assess the dose-relationships with ORP values.

To evaluate the joint effect of plasma metals as a mixture, we simultaneously included all analyzed metals into the elastic net model, which is a shrinkage-based method setting the regularization parameters (λ and α) to prevent the problem of multicollinearity and select an optimal set of ORP-associated metals by cross-validation [46]. In this model, element mixture exposure was calculated as the sum of plasma element levels weighted by their regression coefficients obtained from the elastic net [47]. The associations of metal mixture exposure with ORP values, obstructive CAD, CAD index, and post-PCI outcomes were examined using the models of linear regression, binary logistic regression, ordinal logistic regression, and Cox regression, respectively. We also estimated the C-index, integrated discrimination index (IDI), and net reclassification index (NRI) to assess the independent and incremental values of monitoring metal mixture exposure for prediction of post-PCI outcomes [48]. As an alternative to the elastic net, quantile g-computation (qgcomp) was also performed to yield estimates of mixture effect and associated weights of included metals when simultaneously increasing every metal exposure by 1 quantile [49].

Two sets of progressively adjusted models were constructed for covariate adjustments. Model 1 was adjusted for age, sex, and lifestyle factors (body mass index, cigarette smoking, and alcohol drinking). Model 2 was additionally controlled for physiopathological risk factors (creatinine clearance, C-reactive protein, hypertension, dyslipidemia, peripheral vascular disease, chronic obstructive pulmonary disease, glycated hemoglobin, fasting glucose, diabetes duration, and management of T2DM). Sensitivity analyses were performed by simultaneous adjustment for redox-related metals in the regression models to assess their effects independent of each other and stratified analyses to evaluate the effect modification by baseline characteristics.

We examined the associations of metal mixture exposure with plasma levels of inflammatory cytokines using multiple linear regression (Model 2). We performed a causal mediation analysis to explore the mediating role of inflammatory cytokines in the overall effects of metal mixture on obstructive CAD and post-PCI outcomes, with 5000 bootstrap resampling for internal validation of the mediation effects [50]. To minimize potential impact of extreme outliers of cytokine measurements, the robustness of mediation effects was further tested after excluding participants in the lowest 2.5% and highest 2.5% of cytokine concentrations. We then employed a hierarchical pathway model to estimate overall associations and mediation effects for certain pathways by modeling each cytokines as a linear function of the certain KEGG pathway variables using an inverse-variance weight [36]. Comparisons of *ex vivo* data were made by paired *t*-test or 1-way ANOVA, as appropriate. All statistical analyses were conducted using R (version 4.30)

## 3 Results

### 3.1 Population characteristics

**Fig. 1:**
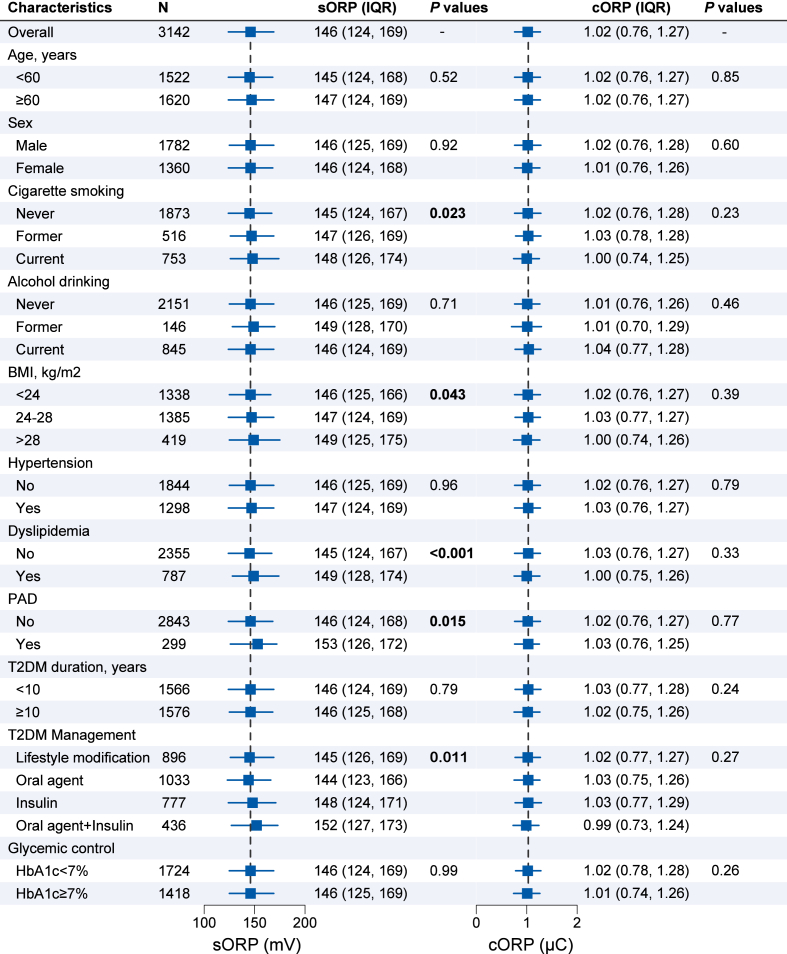
Median (IQR) values of sORP and cORP among type 2 diabetic patients stratified by baseline characteristics. Points represent the median ORP values and lines correspond to the IQR overall and for each subgroup. The dotted line represents the median ORP values in the whole population. *P* values were estimated using the Kruskal-Wallis test (for multi-group comparisons) or the Mann-Whitney *U* test (for 2-group comparisons). ORP indicates oxidation-reduction potential; BMI, body mass index; PAD, peripheral arterial disease; HbA1c, glycated hemoglobin.

In the whole population, the median values of sORP and cORP were 146 (IQR: 124-169 mV) and 1.02 (IQR: 0.76-1.27 μC), respectively. sORP, as an indicator of oxidative stress, was associated positively with the protein oxidation marker protein carbonyl (*P* = 8.7E-5, β = 0.21), the lipid peroxidation marker MDA (*P* = 2.6E-4, β = 0.17), and the oxidative DNA damage marker 8-OHdG (*P* = 1.4E-5, β = 0.35), and negatively with the antioxidant indicators cORP (*P* = 2.3E-5, β = −0.12) and MT (*P* = 1.4E-4, β = −0.35, Supplementary Fig. 2). Plasma sORP values tended to be higher in current smokers, patients with obesity, dyslipidemia, and peripheral arterial disease, and those receiving combination therapy with insulin and oral agents (Fig. 1).

### 3.2 Identification of the redox-related metal mixture

**Fig. 2:**
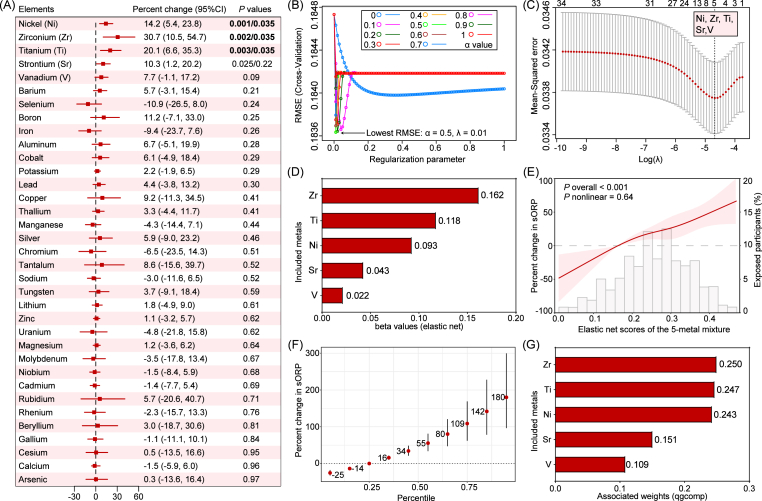
Identification of metal mixture showing strong associations with sORP in type 2 diabetic patients. (**A**) Percent changes in sORP values by plasma levels of 35 elements. (**B** and **C**) The elastic net model setting the regularization parameters for identifying a mixture of 5 sORP-related metals by cross-validation. (**D**) Contributions of individual metals to the mixture effect of 5 sORP-related metals as assessed by the model of elastic net. (**E**) The restricted cubic spline for assessing the dose-response effects of 5 sORP-related metals. (**F**) The quantile g-computation model for assessing the joint effects of 5 sORP-related metals. (**G**) Contributions of individual metals to the mixture effect of 5 sORP-related metals as assessed by the model of quantile g-computation. The models were adjusted for age, sex, body mass index, cigarette smoking, alcohol drinking, creatinine clearance, C-reactive protein, hypertension, dyslipidemia, peripheral vascular disease, chronic obstructive pulmonary disease, glycated hemoglobin, fasting glucose, diabetes duration, and management of diabetes. RMSE indicates root mean square error; qgcomp, quantile g-computation.

Using ICP-MS, we quantified a total of 35 elements, including 17 transition metals, 5 alkali metals, 5 alkaline earth metals, 4 post-transition metals, 2 metalloids, 1 actinoid, and 1 other nonmetal (Supplementary Table 2). After fully controlling for potential confounders (Model 2) and multiple comparisons, plasma levels of nickel, zirconium, and titanium were positively correlated with sORP (Fig. 2A). Comparing the highest with the lowest quartiles of each plasma metal, sORP levels were 38% (95% confidence interval [CI]: 15%-66%) higher for nickel, 28% (95%CI: 7%-35%) higher for zirconium, and 32% (95%CI: 10%-58%) higher for titanium (Supplementary Table 3). In the spline models, the dose-response relationship with sORP was largely linear for each of the 3 metals (Supplementary Fig. 3). The associations were largely consistent when simultaneously including the 3 metals in the regression models (Supplementary Table 4).

To assess the joint effect of metal mixture on oxidative stress, we constructed the best-fit elastic net model (α = 0.5, λ = 0.01, Fig. 2B), which identified a total of 5 metals with non-zero coefficients for predicting sORP, including nickel, zirconium, titanium, strontium, and vanadium (Fig. 2C–D). The 5-metal mixture showed a linear positive correlation with sORP (Fig. 2E), with no effect modification by baseline characteristics (Supplementary Fig. 4). Using the qgcomp model, we further estimated the percent change in sORP as plasma concentrations of all 5 metals were simultaneously fixed at the same percentile. Compared to those with low levels (25th percentile) of metal exposure, patients with high exposure (75th percentile) to the 5-metal mixture had a 109% (95%CI: 62%-169%) increase in sORP (Fig. 2F), with nickel, zirconium, and titanium contributing the most to the association (Fig. 2G). Besides its effects on the overall measure of oxidative stress (i.e. sORP), high exposure to the 5-metal mixture also showed significant associations with increased plasma levels of protein carbonyls, MDA, and 8-OHdG (Supplementary Fig. 5), which are conventional markers of protein, lipid, and DNA oxidation, respectively. Collectively, our data suggest that the 5-metal mixture is closely associated with the global redox status. In contrast, cORP, as a marker of antioxidant reserve, only showed an inverse association with plasma nickel, but not with other metals/metalloids (Supplementary Table 3).

### 3.3 Cross-sectional associations of the redox-related metal mixture with obstructive CAD and CAD extent

**Fig. 3:**
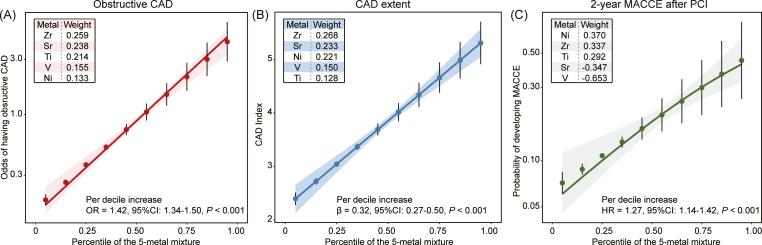
The quantile g-computation models assessing the mixture effects of 5 redox-related metals on obstructive CAD (A), CAD extent (B) and 2-year MACCE after PCI (C) in type 2 diabetic patients. Models were adjusted for age, sex, body mass index, cigarette smoking, alcohol drinking, creatinine clearance, C-reactive protein, hypertension, dyslipidemia, peripheral vascular disease, chronic obstructive pulmonary disease, glycated hemoglobin, fasting glucose, diabetes duration, and management of diabetes. CAD indicates coronary artery disease; MACCE, major adverse cardiovascular and cerebrovascular events.

The causative effects of oxidative stress on cardiovascular disease prompt us to test whether the identified redox-related metal mixture is linked to obstructive CAD. Of the 3142 type 2 diabetic patients, 1470 (47%) patients had obstructive CAD. Compared to those without obstructive CAD, patients with obstructive CAD had increased levels of sORP, protein carbonyl, MDA, 8-OHdG, and GSSG and decreased levels of cORP, MT and GSH (Supplementary Fig. 6), strengthening the linkage of oxidative stress with cardiovascular risk. When compared with the lowest quartile of the redox-related metal mixture as the reference, the fully adjusted OR (Model 2) for obstructive CAD was 1.39 in the second quartile, 1.54 in the third quartile, and 2.53 in the highest quartile (Table 1), with a significant dose-response trend observed (*P*_trend_ < 0.001). Consistent with this observation, the qgcomp model showed linearly increased odds of obstructive CAD with increasing exposure to the redox-related metal mixture (Fig. 3A). When we calculated a 7-category angiographic CAD index to more comprehensively characterize CAD extent across no stenosis, nonobstructive CAD, and obstructive CAD, higher CAD index showed a linear dose-response relationship with increasing percentiles of the redox-related metal mixture (Fig. 3B). Similar results were observed in sensitivity analyses stratified by baseline characteristics (Supplementary Fig. 7). When each metal was individually analyzed, plasma levels of nickel, zirconium, titanium, strontium, and vanadium showed positive relations with obstructive CAD and CAD extent (Table 1 and Supplementary Table 5).

**Table 1:** Cross-sectional associations of redox-related metals and sORP with obstructive CAD and CAD index.

Variables	CAD/Total	Obstructive CAD		CAD index	
		Model 1^a^	Model 2^b^	Model 1^a^	Model 2^b^
5-metal mixture					
Q1 (<0.19)	282/786	1 (reference)	1 (reference)	1 (reference)	1 (reference)
Q2 (0.19-0.26)	348/785	1.38 (1.12-1.69)	1.39 (1.13-1.72)	1.27 (1.06-1.51)	1.26 (1.06-1.51)
Q3 (0.26-0.32)	371/787	1.55 (1.26-1.90)	1.54 (1.25-1.89)	1.45 (1.21-1.72)	1.42 (1.19-1.70)
Q4 (>0.32)	469/784	2.51 (2.04-3.08)	2.53 (2.05-3.12)	1.91 (1.61-2.28)	1.88 (1.58-2.25)
Per quartile increase		1.33 (1.25-1.42)	1.33 (1.25-1.43)	1.23 (1.16-1.30)	1.22 (1.16-1.29)
Nickel (μg/L)					
Q1 (<2.39)	340/786	1 (reference)	1 (reference)	1 (reference)	1 (reference)
Q2 (2.40-4.18)	347/785	1.02 (0.83-1.25)	1.03 (1.03-1.26)	1.08 (0.90-1.28)	1.08 (0.90-1.28)
Q3 (4.19-5.92)	367/785	1.10 (0.90-1.35)	1.11 (0.90-1.36)	1.17 (0.98-1.39)	1.18 (0.99-1.40)
Q4 (>5.92)	416/786	1.37 (1.12-1.69)	1.37 (1.12-1.69)	1.30 (1.09-1.54)	1.27 (1.07-1.52)
Per quartile increase		1.11 (1.04-1.18)	1.11 (1.04-1.18)	1.09 (1.03-1.15)	1.08 (1.03-1.15)
Zirconium (μg/L)					
Q1 (<0.09)	340/785	1 (reference)	1 (reference)	1 (reference)	1 (reference)
Q2 (0.09-0.12)	329/790	0.95 (0.78-1.17)	0.94 (0.77-1.16)	0.96 (0.81-1.15)	0.96 (0.80-1.14)
Q3 (0.12-0.16)	348/783	1.05 (0.85-1.28)	1.06 (0.86-1.30)	1.07 (0.90-1.27)	1.08 (0.90-1.28)
Q4 (>0.16)	453/784	1.81 (1.48-2.22)	1.85 (1.51-2.28)	1.53 (1.28-1.82)	1.55 (1.30-1.84)
Per quartile increase		1.21 (1.13-1.29)	1.22 (1.14-1.30)	1.15 (1.09-1.21)	1.15 (1.09-1.22)
Titanium (μg/L)					
Q1 (<19.34)	339/785	1 (reference)	1 (reference)	1 (reference)	1 (reference)
Q2 (19.34-31.58)	356/786	1.06 (0.87-1.30)	1.09 (0.89-1.34)	1.07 (0.90-1.27)	1.09 (0.92-1.30)
Q3 (31.59-42.75)	343/787	1.00 (0.82-1.22)	1.00 (0.82-1.23)	0.98 (0.82-1.17)	0.99 (0.83-1.18)
Q4 (>42.75)	432/784	1.54 (1.26-1.89)	1.55 (1.26-1.91)	1.33 (1.12-1.58)	1.33 (1.11-1.58)
Per quartile increase		1.13 (1.06-1.21)	1.13 (1.06-1.21)	1.08 (1.02-1.14)	1.08 (1.02-1.14)
Strontium (μg/L)					
Q1 (<19.50)	341/790	1 (reference)	1 (reference)	1 (reference)	1 (reference)
Q2 (19.50-35.28)	321/783	0.92 (0.75-1.13)	0.92 (0.75-1.13)	0.93 (0.78-1.10)	0.92 (0.77-1.10)
Q3 (35.29-52.30)	345/784	1.02 (0.83-1.24)	1.01 (0.82-1.24)	0.97 (0.82-1.16)	0.96 (0.81-1.15)
Q4 (>52.30)	463/785	1.88 (1.54-2.31)	1.87 (1.52-2.30)	1.65 (1.39-1.96)	1.62 (1.36-1.92)
Per quartile increase		1.22 (1.14-1.30)	1.22 (1.14-1.30)	1.17 (1.10-1.23)	1.16 (1.10-1.23)
Vanadium (μg/L)					
Q1 (<1.34)	341/785	1 (reference)	1 (reference)	1 (reference)	1 (reference)
Q2 (1.34-2.54)	343/786	1.01 (0.82-1.23)	1.00 (0.81-1.22)	1.01 (0.85-1.20)	1.00 (0.84-1.19)
Q3 (2.55-3.73)	330/787	0.93 (0.76-1.14)	0.94 (0.77-1.16)	0.93 (0.78-1.11)	0.93 (0.78-1.11)
Q14 (>3.73)	456/784	1.81 (1.48-2.22)	1.79 (1.46-2.21)	1.65 (1.38-1.97)	1.62 (1.36-1.93)
Per quartile increase		1.19 (1.11-1.27)	1.19 (1.11-1.27)	1.15 (1.09-1.22)	1.15 (1.09-1.21)
sORP (mV)					
Q1 (<125)	329/790	1 (reference)	1 (reference)	1 (reference)	1 (reference)
Q2 (125-146)	353/789	1.17 (0.95-1.43)	1.20 (0.98-1.48)	1.14 (0.96-1.36)	1.17 (0.98-1.40)
Q3 (147-169)	350/804	1.11 (0.91-1.36)	1.07 (0.87-1.32)	1.11 (0.94-1.33)	1.08 (0.90-1.28)
Q4 (>169)	438/759	1.87 (1.53-2.30)	1.91 (1.55-2.35)	1.57 (1.31-1.87)	1.56 (1.31-1.87)
Per quartile increase		1.20 (1.12-1.28)	1.20 (1.12-1.28)	1.14 (1.08-1.21)	1.13 (1.07-1.20)

Q indicates quartile; CAD, coronary artery disease; sORP, static oxidation-reduction potential.

a Model 1 was adjusted for age, sex, body mass index, cigarette smoking, and alcohol drinking.

b Model 2 was adjusted for age, sex, body mass index, cigarette smoking, alcohol drinking, creatinine clearance, C-reactive protein, hypertension, dyslipidemia, peripheral vascular disease, chronic obstructive pulmonary disease, glycated hemoglobin, fasting glucose, diabetes duration, and management of diabetes.

To test whether high exposure to the redox-related metal mixture was observed in all type 2 diabetic patients or only in those with obstructive CAD, we further compared the elemental profiles of 3142 type 2 diabetic patients with 1000 nondiabetic subjects. Generally, plasma concentrations of the 5 redox-related metals were gradually increased in nondiabetic subjects, T2DM patients without obstructive CAD, and T2DM patients with obstructive CAD (Supplementary Table 6).

### 3.4 Prospective associations of the redox-related metal mixture with 2-year clinical outcomes after PCI

Of patients with obstructive CAD, 1212 patients underwent primary PCI within 30 days after angiography. During a median follow-up of 23.8 months (IQR: 23.1-24.4 months), a total of 236 (19.5%) MACCEs were identified. Generally, high exposure to the redox-related metal mixture linearly increased the probability of developing MACCE at 2 years after PCI (Fig. 3C). The corresponding hazard ratios were 1.38 (95%CI: 1.22-1.55) per 1-quartile increase in the redox-related metal mixture and 2.44 (95%CI: 1.65-3.59) comparing the highest with the lowest quartiles of the redox-related metal mixture (Model 2 in Table 2). When analyzed individual components of MACCEs as the outcomes, the hazards of all-cause death, cardiovascular death, and repeat revascularization increased across quartiles of the redox-related metal mixture (Supplementary Fig. 8). When parameters of metals and ORPs were individually analyzed, plasma levels of nickel, zirconium, and titanium plus sORP showed significantly positive associations with post-PCI outcomes (Table 2 and Supplementary Table 7). When the redox-related metal mixture and sORP were fitted into the SYNTAX II score, the discrimination and net reclassification of MACCE risk were also significantly improved (Supplementary Table 8).

**Table 2:** Prospective associations of redox-related metals and sORP with 2-year MACCE after PCI.

Variables	Events/Total	Model 1^a^	Model 2^b^
5-metal mixture			
Q1	37/303	1 (reference)	1 (reference)
Q2	44/303	1.18 (0.76-1.82)	1.17 (0.75-1.81)
Q3	68/303	1.91 (1.28-2.85)	1.93 (1.29-2.88)
Q4	87/303	2.48 (1.68-3.65)	2.44 (1.65-3.59)
Per quartile increase		1.38 (1.23-1.56)	1.38 (1.22-1.55)
Nickel (μg/L)			
Q1	45/303	1 (reference)	1 (reference)
Q2	50/303	1.12 (0.75-1.67)	1.14 (0.76-1.71)
Q3	62/303	1.42 (0.97-2.08)	1.44 (0.98-2.11)
Q4	79/303	1.85 (1.28-2.68)	1.91 (1.32-2.77)
Per IQR		1.24 (1.10-1.39)	1.25 (1.11-1.41)
Zirconium (μg/L)			
Q1	46/303	1 (reference)	1 (reference)
Q2	52/305	1.13 (0.76-1.68)	1.11 (0.74-1.65)
Q3	62/302	1.41 (0.96-2.07)	1.37 (0.93-2.00)
Q4	76/302	1.78 (1.23-2.57)	1.75 (1.21-2.53)
Per quartile increase		1.22 (1.09-1.37)	1.21 (1.08-1.36)
Titanium (μg/L)			
Q1	40/303	1 (reference)	1 (reference)
Q2	58/304	1.44 (0.96-2.16)	1.43 (0.95-2.14)
Q3	62/302	1.52 (1.02-2.26)	1.45 (0.97-2.17)
Q4	76/303	1.90 (1.29-2.80)	1.87 (1.27-2.76)
Per quartile increaseR		1.21 (1.08-1.36)	1.20 (1.07-1.36)
Strontium (μg/L)			
Q1	64/304	1 (reference)	1 (reference)
Q2	43/302	0.64 (0.44-0.95)	0.65 (0.44-0.96)
Q3	70/303	1.06 (0.75-1.48)	1.09 (0.77-1.53)
Q4	59/303	0.87 (0.61-1.23)	0.88 (0.62-1.26)
Per quartile increase		1.01 (0.89-1.13)	1.01 (0.90-1.13)
Vanadium (μg/L)			
Q1	59/304	1 (reference)	1 (reference)
Q2	63/301	1.10 (0.77-1.57)	1.09 (0.76-1.56)
Q3	55/303	0.95 (0.66-1.37)	0.95 (0.65-1.37)
Q4	59/304	1.03 (0.72-1.48)	1.01 (0.71-1.46)
Per quartile increase		0.99 (0.89-1.11)	0.99 (0.88-1.11)
sORP (mV)			
Q1	36/312	1 (reference)	1 (reference)
Q2	53/300	1.56 (1.02-2.39)	1.54 (1.00-2.35)
Q3	66/297	2.06 (1.37-3.09)	2.11 (1.41-3.18)
Q4	81/303	2.44 (1.65-3.62)	2.45 (1.65-3.65)
Per quartile increase		1.32 (1.18-1.49)	1.33 (1.18-1.50)

Q indicates quartile; sORP, static oxidation-reduction potential.

a Model 1 was adjusted for age, sex, body mass index, cigarette smoking, and alcohol drinking.

b Model 2 was adjusted for age, sex, body mass index, cigarette smoking, alcohol drinking, creatinine clearance, C-reactive protein, hypertension, dyslipidemia, peripheral vascular disease, chronic obstructive pulmonary disease, glycated hemoglobin, fasting glucose, diabetes duration, and management of diabetes.

### 3.5 Mediating roles of plasma cytokines in the effects of the redox-related metals

**Fig. 4:**
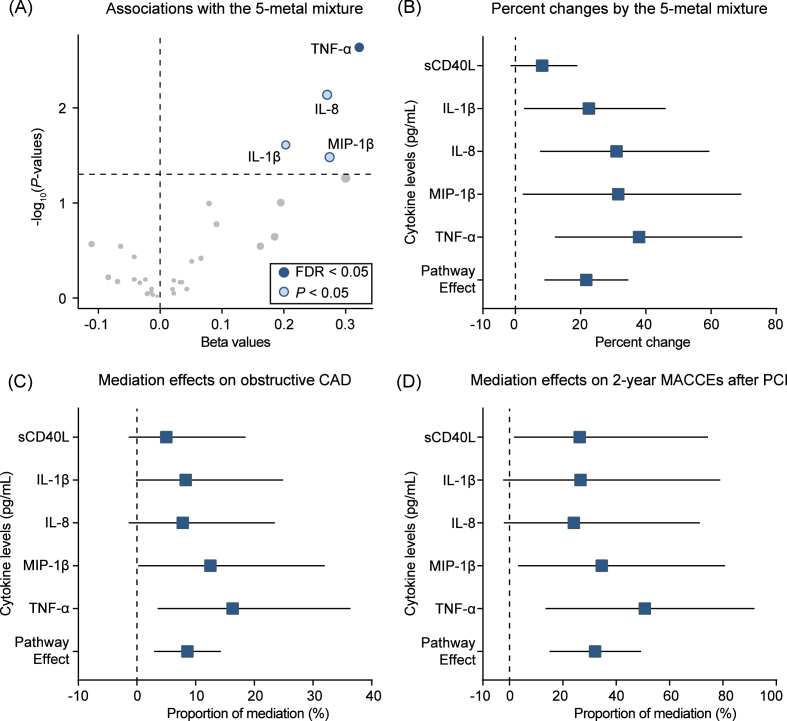
Increased concentrations of cytokines within the NF-κB pathway mediate the associations of the redox-related metal mixture with obstructive CAD and post-PCI outcomes. (**A**) The volcano plot depicting the associations of the redox-related metal mixture with plasma concentrations of 31 cytokines. Dark blue dots indicate significant associations with FDR <0.05; Light blue dots indicate nominal associations with *P* < 0.05. (**B**) Percent changes in cytokines within the NF-κB pathway by the redox-related metal mixture. (**C**) Mediation effects of cytokines within the NF-κB pathway on the cross-sectional association between the redox-related metal mixture and obstructive CAD. (**D**) Mediation effects of cytokines within the NF-κB pathway on the prospective association between the redox-related metal mixture and 2-year MACCE after PCI. Bootstrap with 5000 resampling was performed to estimate the effect sizes and confidence intervals of the mediation effects. Models were adjusted for age, sex, body mass index, cigarette smoking, alcohol drinking, creatinine clearance, C-reactive protein, hypertension, dyslipidemia, peripheral vascular disease, chronic obstructive pulmonary disease, glycated hemoglobin, fasting glucose, diabetes duration, and management of diabetes.

Considering that inflammation is a common mechanism linking metal exposure and oxidative stress to cardiovascular health, we further performed a cytokine profiling analysis to test whether inflammatory cytokines mediate the adverse effects of the redox-related metals. Generally, after controlling for confounding factors and multiple comparisons, high exposure to the redox-related metal mixture showed significant associations (FDR <0.05) with increased concentrations of the cytokine tumor necrosis factor α and nominal associations (*P* < 0.05) with increased concentrations of interleukin (IL) 1β, IL-8, and macrophage inflammatory protein 1β (Fig. 4A and Supplementary Table 9). Importantly, the hierarchical pathway analysis demonstrated that high exposure to the 5-metal mixture was associated with an average increase of 22% (95%CI: 9%-35%, *P* = 0.001, Fig. 4B) in cytokines within the NF-κB pathway. Using cytokines as the mediators, the causal mediation analysis showed that cytokines within the NF-κB pathway significantly mediated 9% (95%CI: 3%-14%, *P* = 0.003, Fig. 4C) of the association between the redox-related metal mixture and obstructive CAD. When analyzed post-PCI events as the outcome, up to 32% (95%CI: 15%-49%, *P* < 0.001, Fig. 4D) of the total effects of the redox-related metal mixture on 2-year MACCEs after PCI could be explained via cytokines within the NF-κB pathway. The mediation effects remained significant after excluding extreme outliers of cytokine measurements (Supplementary Fig. 9).

### 3.6 Ex vivo models characterizing the bioenergetic consequences of the redox-related metal exposure in PBMCs

**Fig. 5:**
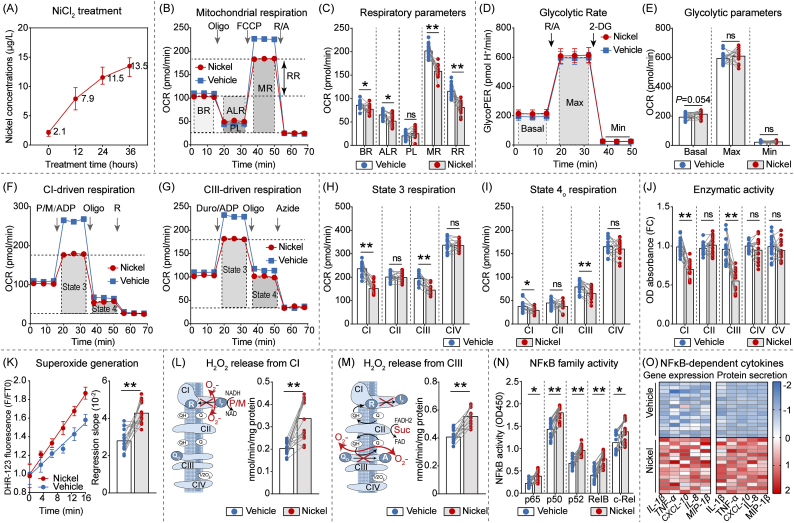
Exposure to nickel induces a specific pattern of bioenergetic dysfunction characterized by mitochondrial respiratory impairment, mtROS overproduction and NF-κB activation in PBMCs. (A) Time-dependent effects of NiCl_2_ treatment on concentrations of nickel in PBMCs. (**B** and **C**) Effects of nickel exposure on the overall capacity of mitochondrial respiration. (**D** and **E**) Effects of nickel exposure on the overall capacity of glycolysis. (**F** and **G**) Effects of nickel exposure on the respiratory activities of CI and CIII. (**H** and **I**) Summarized results for the effects of nickel exposure on the rates of state 3 and state 4 respiration driven by CI to CIV. (**J**) Summarized results for the effects of nickel exposure on the enzymatic activities of the ETC complexes (CI to CV). (**K**) Effects of nickel exposure on mitochondrial superoxide generation. (**L** and **M**) Effects of nickel exposure on CI- and CIII-driven H_2_O_2_ release; (**N**) Effects of nickel exposure on the activities of the NF-κB family; (**O**) Effects of nickel exposure on mRNA expression and protein secretion of NF-κB-dependent cytokines. N = 15 for each group. *P < 0.05; **P < 0.001; ns, nonsignificant. Oligo indicates oligomycin; R, rotenone; A, Antimycin A; BR, basal respiration; ALR, ATP-linked respiration; PL, proton leak; MR, maximal respiration; RR, reserve respiration; 2-DG, 2-deoxy-d-glucose; P, pyruvate; M, malate; Duro, duroquinol; Suc, succinate.

To characterize the bioenergetic consequences under exposure to redox-related metals, we created *ex vivo* models of PBMCs with high exposure to nickel, titanium, and zirconium (the top 3 redox-related metals). As presented in Fig. 5A, treatment with 50 ng/mL NiCl_2_⋅6H_2_O for 24 h substantially increased nickel concentrations in PBMCs from 2.1 μg/L to 11.5 μg/L, a level close to that in nickel-exposed subjects [51]. This exposure dose had no significant effects on cell viability, apoptosis, and mitochondrial content (as indicated by citrate synthase activity, Supplementary Fig. 10). Using the Mito Stress test, we performed a detailed analysis of mitochondrial respiration, which showed decreased rates of basal, ATP-linked, maximal, and reserve respiration in nickel-exposed PBMCs (Fig. 5B and C). In parallel, measurements of glycolytic flux revealed a subtle increase (+10%, *P* = 0.054) in baseline glycolysis by nickel exposure. Importantly, when rotenone and antimycin A were subsequently injected to completely block the mitochondrial respiratory chain, the induced maximum glycolysis did not change by nickel exposure (Fig. 5D and E), suggesting that the nickel-mediated increase in baseline glycolysis is not due to an enhancement of maximal glycolytic capacity, but to a compensatory response of mitochondrial respiratory impairment.

To seek for the end-effectors of nickel-mediated impairment of mitochondrial respiration, we further measured the respiratory rates driven by individual respiratory complexes in permeabilized PBMCs. Overall, boron exposure induced substantial decreases in ADP-stimulated state 3 respiration and oligomycin-stimulated state 4 respiration when adding the substrates for CI and CIII, but not the substrates for CII and CIV (Fig. 5F–I). These respirometric results concur with the colorimetric data, which showed significant reductions in enzymatic activities of CI and CIII in nickel-exposed PBMCs (Fig. 5J). Considering that CI and CIII are the 2 major producers of mtROS [43], we further used the DHR123 probe to measure the rate of mitochondrial superoxide production, which was indeed upregulated by nickel exposure (Fig. 5K). Importantly, by adding CI- and CIII-linked substrates, we observed a marked elevation of mitochondrial H_2_O_2_ release in nickel-exposed PBMCs (Fig. 5L and M), validating CI and CIII as the major contributors to nickel-induced mtROS production. Notably, the nickel-induced upregulation of mtROS was accompanied by downregulation of GSH and NADPH (Supplementary Fig. 11), suggesting a compromise of antioxidant systems in nickel-exposed PBMCs.

Since mtROS have been widely reported as the key activators of the NF-κB pathway [52], we explored the role of NF-κB activation in connecting mitochondrial respiratory impairment and inflammatory cytokine production. Indeed, exposing PBMCs to nickel greatly enhanced the DNA-binding activity of the NF-κB subunits (Fig. 5N), accompanied by upregulation of NF-κB-dependent proinflammatory cytokine gene expression and protein secretion (Fig. 5O). Notably, treatment with MitoQ (0.5 μM for 24 h), a mitochondria-specific ROS scavenger, in the presence of nickel, did not change mitochondrial respiration, but greatly inhibited NF-κB activation and subsequent proinflammatory cytokine gene expression. In contrast, pharmacological inhibition of NF-κB by BAY 11-7082 (2.5 μM for 24 h) substantially mitigated the nickel-induced surge on NF-κB-dependent proinflammatory cytokine gene expression, but failed to suppress mtROS production (Supplementary Fig. 12). Collectively, our data demonstrate that nickel-induced respiratory impairment causes simultaneous mtROS generation, which mediates NF-κB-dependent proinflammatory cytokine production in PBMCs.

Importantly, when PBMCs were exposed to titanium and zirconium by treating with 100 ng/mL titanium dioxide nanoparticles and 10 ng/mL zirconia nanoparticles, respectively, we observed a similar pattern of bioenergetic dysfunction characterized by impaired respiration of CI and CIII, mtROS overproduction, NF-κB activation, and NF-κB-dependent cytokine expression (Supplementary Figs. 13–14), suggesting that such pattern is a common bioenergetic state in PBMCs exposed to redox-related metals.

## 4 Discussion

ORP is a comprehensive measure of oxidation-reduction equilibrium, which has been introduced to evaluate the global redox status in human biological fluids [53]. Although some small studies have suggested sORP as a potential predictor of redox-related diseases including metabolic syndrome and male infertility [12,54], evidence for the use of the ORP measurement to reflect the metal-mediated oxidative stress is still scarce. Here, in a large cohort of 3142 type 2 diabetic patients, our study integrated elementomics approach and ORP measurement to identify a mixture of 5 metals with significant effects on global redox status. Importantly, this 5-metal mixture showed a strong association not only with sORP, but with validated markers of protein, lipid, and DNA oxidation, strengthening the robustness of the identification of redox-related metals using sORP.

Accumulating evidence for the causal link between oxidative stress and cardiovascular disease prompts us to further test whether sORP is a novel marker of cardiovascular events. By systematically evaluating the continuum of CAD extent and tracking patients’ longitudinal post-PCI outcomes, our study indeed showed that higher values of plasma sORP cross-sectionally correlated with obstructive CAD and prospectively predicted adverse outcomes after PCI. This finding from our hypothesis-driven analyses was consistent with results from machine learning approaches for the linear relationships with both cardiovascular endpoints when the 5 redox-related metals were jointly analyzed, supporting that the associations between sORP and metal exposure are clinically relevant to cardiovascular events. Furthermore, by profiling plasma cytokines, our study shed new lights on potential mechanism linking redox-related metals and cardiovascular health by characterizing the mediating roles of proinflammatory cytokines within the NF-κB pathway in the mixture effects of the 5 redox-related metals on CAD risk and post-PCI outcomes.

Of the 5 redox-related metals, nickel, zirconium, and titanium contributed the most to the overall association with the sORP-indicated global redox status. Nickel, as a natural element present in sulfide/oxide ores, is extensively used in the production of everyday objects, as well as in medical applications including vascular stents and prothesis implants [55]. Experimental studies have consistently reported that nickel exposure is functionally linked to increased ROS production in multiples cells and tissues [56]. The current study extends previous work by revealing a novel association between plasma nickel and the oxidative stress marker sORP in a diabetic population. Furthermore, our *ex vivo* experiments provide a deeper understanding of the detrimental roles of nickel exposure in bioenergetics of PBMCs. First, by in-depth analyses of respiratory activities driven by the respiratory chain and the individual ETC complexes, we delineated the bioenergetic consequences of nickel exposure characterized by substantial reductions of CI- and CIII-driven respiratory activities, which led to the full-scale downregulation of basal, ATP-linked, maximal, and reserve respiration in PBMCs. Then, by comprehensively modeling superoxide generation in intact PBMCs and H_2_O_2_ release in isolated mitochondria, we obtained consistent results indicating that the nickel-induced respiratory impairment is a pivotal pathological event driving mtROS release at CI and CIII. Further, focusing on the interdependence between ROS production and inflammatory status, our data suggest that the effects of nickel exposure on promoting ROS production may represent an upstream mechanism triggering NF-κB activation and consequently mediating NF-κB-dependent proinflammatory cytokine generation. Together, our findings may establish a mechanistic link between nickel exposure, ROS production, and NF-κB inflammation pathway. Of note, our study also observed positive relationships between plasma nickel and cardiovascular outcomes (i.e. increased CAD risk and poorer post-PCI outcomes), which were generally consistent with previous studies reporting positive associations of environmental nickel exposure with cardiovascular events and mortality [57,58], highlighting the adverse effects of nickel exposure on cardiovascular health.

Globally, zirconium has increasingly been used as an alternative to traditional heavy metals in industrial processes, yet the epidemiological data on its health effects remain sparse. Our study is the first to report significant associations of zirconium exposure with higher extent of oxidative stress, increased risk of obstructive CAD, and poor post-PCI outcomes. The plasma concentrations of zirconium in our study population were relatively low, ranging from 0.05 μg/L to 0.21 μg/L. Such low concentrations were in contrast to the high detection rates of zirconium, suggesting that our study participants were generally exposed to low levels of zirconium. However, a recent biomonitoring study has shown that zirconium has the unique biokinetic features of high tissue retention, slow clearance, and limited urinary excretion, which determine that zirconium exposure, even at sub-threshold levels, may still produce subclinical effects on hematological parameters and pulmonary function in humans [59]. In light of this, we performed *ex vivo* experiments by exposing PBMCs to low-level zirconium, which still induced detrimental effects on respiratory, redox, and inflammatory states of PBMCs. These findings, combined with previous animal models showing severe oxidative damage induced by zirconia nanoparticles [60], pinpoint the urgent need for more health concerns related to zirconium, even at low exposure doses.

Due to its high strength and corrosion resistance, titanium is usually alloyed with other metals in industrial manufacturing and medical applications. Although titanium has high biocompatibility, its cytotoxic effect, as a direct result of the induction of oxidative stress, has been increasingly reported in toxicity studies. Recently, a nested case-control study has observed a positive association between plasma titanium and incident coronary heart disease in retired auto manufacturing workers, raising concerns about the adverse health effects of titanium exposure [61]. Here, in type 2 diabetic patients, higher levels of plasma titanium also showed a cross-sectional association with obstructive CAD and a prospective association with poorer post-PCI outcomes. This finding is meaningful because the consistent associations across populations with different background risk factors suggest that titanium exposure is linked to cardiovascular health independent of confounding risk factors [62]. Importantly, our *ex vivo* experiments in PBMCs further showed that titanium exposure induced a pattern of bioenergetic dysfunction similar to nickel and zirconium exposures. These results concur with the summarized toxicity data for multiple metals, which support the notion that redox-related metals may share common mechanisms (such as the Fenton reaction) to cause oxidative damage [63].

Previous experimental studies have provided strong evidence for the link between iron overload and oxidative stress, especially under diabetic conditions [64]. However, epidemiological studies attempting to demonstrate this link using total iron or ferritin as markers of iron status have been generally negative [65]. Our study using ICP-MS to measure total plasma iron also did not find significant associations with the oxidative stress marker sORP and cardiovascular outcomes in type 2 diabetic patients. Physiologically, most of the iron in the circulation is sequestered in the transport protein, ferritin, in which iron is redox-inactive. Thus, methods for measuring total plasma iron (such as ICP-MS) mainly target redox-inactive iron. While for type 2 diabetic patients, the molecular nature of catalytically redox active iron may indeed exist in aberrantly glycated proteins with an increased affinity for iron [66]. So, measuring non-transferrin bound, redox-active iron rather than total iron status is more likely to reflect the risk of iron-mediated oxidative injury [67].

The main strengths of our study are the application of the novel oxidative stress marker ORP for comprehensive identification of redox-related metals, with the use of both cross-sectional and prospective designs for cardiovascular risk assessment, the profiling of proinflammatory cytokines to gain insight into potential underlying pathways, and the detailed characterization of respiratory, redox, and inflammatory changes induced by redox-related metals in PBMCs. Nevertheless, our study still has potential limitations. First, although we performed multiple sensitivity analyses to control for confounding, residual confounding cannot be ruled out due to the observational design. Second, we relied on a single baseline measurement of plasma metals, which may not capture long-term exposure to metals with short half-lives. Third, although plasma samples have been widely used and validated to reflect the exposure status for most metals [61], they may not be suitable for assessment of internal exposure for some metals (such as arsenic and lead). Fourth, we quantified the markers of protein, lipid, and DNA peroxidation using the colorimetric and ELISA assays, which are more practicable in epidemiological studies but may have relatively low specificity and sensitivity compared with HPLC or MS-based methods. Finally, our study focused on the identification of redox-related metals in type 2 diabetic patients, which may limit the generalizability of our findings to nondiabetic subjects.

## 5 Conclusions

Using ORP as an indicator of global redox status, our study identified a mixture of 5 redox-related metals including nickel, zirconium, titanium, strontium, and vanadium in patients with T2DM. Both elevated values of sORP and high exposure to the redox-related metal mixture showed cross-sectional associations with obstructive CAD and prospective associations with poor outcomes after PCI. Subsequent mediation analyses revealed that changes in NF-κB-dependent cytokines might mediate these associations. Further *ex vivo* experiments in PBMCs delineated a pattern of bioenergetic dysfunction induced by redox-related metals, which involved CI- and CIII-linked respiratory impairment, mtROS overproduction, and NF-κB-dependent cytokine expression. These findings indicate sORP as a promising marker of metal-induced oxidative stress and support the links of redox-related metals to cardiometabolic risk in patients with T2DM.

## Funding

This study was supported by the Henan Clinical Medical Scientist Program (HNCMS202526), the 10.13039/501100001809National Natural Science Foundation of China (82570405 and 82170343), the 10.13039/501100006407Natural Science Foundation of Henan Province for Excellent Young Scholars (242300421087), and the Young and Middle-Aged Health Science and Technology Innovation Talent of Henan Province (JQRC2024007).

## CRediT authorship contribution statement

**Zi-Qi Fang:** Data curation, Investigation, Methodology. **Bing Wang:** Data curation, Investigation, Methodology. **Ning-Hua Cui:** Methodology, Software, Visualization. **Zhe-Zhe Yang:** Methodology, Software, Visualization. **Xue-Bin Wang:** Conceptualization, Funding acquisition, Writing – original draft, Writing – review & editing.

## Declaration of competing interest

The authors do not have anything to disclose regarding conflict of interest with respect to this manuscript.
