# Supplementary material for: An integrative analysis of elementomics and oxidation-reduction potentials identifies redox-related metals associated with coronary artery disease and post-PCI outcomes in type 2 diabetic patients
Source: Redox Biol. 2026 Jun 19;95:104270. doi: 10.1016/j.redox.2026.104270 (PMC13314796; doi:10.1016/j.redox.2026.104270)
Supplement: Multimedia component 1 [file mmc1.pdf]

SUPPLEMENTARY MATERIALS

An integrative analysis of elementomics and oxidation-reduction potentials identifies redox-related metals associated with coronary artery disease and post-PCI outcomes in type 2 diabetic patients

Correspondence: Xue-Bin Wang

Department of Clinical Laboratory, Key Clinical Laboratory of Henan Province, The First Affiliated Hospital of Zhengzhou University, Jianshe East Road No.1, Zhengzhou 450000, Henan, China. E-mail: xbwang2017@163.com

The Supplementary Materials includes:

Supplementary Methods

Supplementary Tables 1 to 9

Supplementary Figures 1 to 14

Catalogue

Supplementary Methods **3**

1. Baseline and outcome data 3
2. Elementomics by inductively coupled plasma-mass spectrometry 6
3. *Ex vivo* experiments 7

Supplementary Tables **11**

Supplementary Table 1 11

Supplementary Table 2 12

Supplementary Table 3 13

Supplementary Table 4 18

Supplementary Table 5 19

Supplementary Table 6 20

Supplementary Table 7 21

Supplementary Table 8 22

Supplementary Table 9 23

Supplementary Figures **24**

Supplementary Fig. 1 24

Supplementary Fig. 2 25

Supplementary Fig. 3 26

Supplementary Fig. 4 27

Supplementary Fig. 5 28

Supplementary Fig. 6 29

Supplementary Fig. 7 30

Supplementary Fig. 8 31

Supplementary Fig. 9 32

Supplementary Fig. 10 33

Supplementary Fig. 11 34

Supplementary Fig. 12 35

Supplementary Fig. 13 36

Supplementary Fig. 14 37

Supplementary Methods

1. Baseline and outcome data

1.1 Definition of baseline characteristics

Type 2 diabetes mellitus (T2DM) was diagnosed based on the 2014 criteria of the American Diabetes Association: fasting glucose ≥ 7.0 mM or 2-h post-load glucose levels ≥ 11.1 mM or glycated hemoglobin ≥ 6.5% or ongoing therapy for T2DM [1].

Current smokers were defined as having smoked ≥ 100 cigarettes in their lifetime and now smoking every day or some days. Ever smokers were defined as having quitted smoking at least 1 year prior to interview. Subjects with alcohol consumption at least once a week for ≥ 1 year were defined as “alcohol drinkers”. One drink was defined as 375 ml of beer (13.6 g of ethanol), 118 ml of wine (11.7 g of ethanol), or 30 ml of western or Chinese hard liquor (10.9 g of ethanol). Hypertension was defined as ongoing therapy for hypertension, systolic blood pressure of ≥ 140mmHg or diastolic blood pressure of ≥ 90mmHg. Dyslipidemia was defined as hypercholesterolemia (total cholesterol > 5.72 mM), high levels of low-density lipoprotein cholesterol (> 3.1 mM), low levels of high-density lipoprotein cholesterol (< 0.9 mM), hypertriglyceridemia (triglycerides > 1.70 mM). Peripheral vascular disease (PAD) was defined as arteries other than coronaries, with exercise-related claudication, revascularization surgery, reduced or absent pulsation, and/or angiographic stenosis of > 50%. Chronic obstructive pulmonary disease (COPD) was defined as the long-term use of bronchodilators or steroids for lung disease.

Weight and height were measured during baseline examinations. Fasting glucose,

triglycerides, total cholesterol, low-density lipoprotein cholesterol, high-density lipoprotein cholesterol, and C-reactive protein were measured on a Cobas 8000 Analyzer (Roche Diagnostics, Germany) using standard methods. Glycosylated haemoglobin was assayed by high-performance liquid chromatography on a Bio-Rad Hemoglobin Testing System.

1.2 Definition of cross-sectional outcomes

At baseline, all participants underwent invasive coronary angiography using the Judkins percutaneous trans-femoral technique. Digital angiograms were reviewed by two expert observers to document lesion characteristics. Obstructive coronary artery disease (CAD) was defined as coronary artery stenoses of > 50% in at least 1 major epicardial coronary arteries. The anatomical complexity of CAD was assessed using the SYNTAX score [2]. The severity of coronary stenosis was evaluated using an angiographic CAD index, which created 7 categories of CAD severity: no apparent CAD (< 20% stenosis); 1-, 2-, and 3-vessel nonobstructive CAD (20%-50% stenosis); 1-, 2-, and 3-vessel obstructive CAD (> 50% stenosis), based on the extent of maximal stenosis and the number of diseased vessels.

1.3 Indications for percutaneous coronary intervention (PCI)

Indications for primary PCI after coronary angiography included suitable anatomy for PCI plus or/and high-risk clinical presentations (including dynamic electrocardiogram changes, elevated cardiac markers, and high Global Registry of Acute Coronary Events scores) or/and recurrent symptoms (defined as recurrent angina or ischemia at rest or with low-level activities despite intensive medical therapy) or/and

new or presumably new ST-segment depression or/and signs of large ischemic burden (including symptoms of heart failure, left ventricular ejection fraction < 40%, hemodynamically comprising ventricular tachycardia, and cardiogenic shock) [3]. Delayed PCI after angiography might apply to 1) patients with stable ischemic heart disease (SIHD) with ≥ 1 significant stenoses amenable to PCI and unacceptable angina despite guideline-directed medical therapy (GDMT) and 2) patients with SIHD with unacceptable angina in whom GDMT cannot be continued due to adverse effects or patient preferences [4].

1.4. Definitions of post-PCI outcomes

Of the 3142 participants, 1212 who received primary PCI within 30 days after angiography completed a 2-year follow-up survey for prospectively tracking post-PCI outcomes. Outcome data were documented through telephone interviews with patients and medical record reviews at 30 days, 1 year, and 2 years after PCI, and centrally adjudicated by 2 cardiologists who were blinded to the baseline data.

The primary outcome was major adverse cardiovascular and cerebrovascular events (MACCE), a patient-oriented composite endpoint composed of all-cause death, non-fatal myocardial infarction, non-fatal stroke, and repeat revascularization. All-cause death was defined as death from any cause. Cardiovascular death was defined as death due to myocardial infarction, stroke, significant cardiac arrhythmia, refractory congestive heart failure, or other specified cardiovascular causes. Myocardial infarction was defined as ischaemic signs or symptoms and new pathological Q-waves in ≥ 2 contiguous ECG leads, or/and an elevation CK-MB or troponin above the 99th

percentile limit of normal and at least ≥ 20% above the most recent value. Stroke was defined as a focal neurologic deficit of central origin lasting > 72 hours, or a focal neurologic deficit of central origin lasting > 24 hours, with imaging evidence of cerebral infarction or intracerebral hemorrhage. Repeat revascularization was defined as any repeat PCI. All stages of a staged index PCI procedure would be considered part of the index revascularization procedure and not a repeat revascularization.

2. Elementomics by inductively coupled plasma-mass spectrometry

2.1. Sample preparation

For all participants, venous blood samples were drawn before angiography. Plasma samples were collected and stored at -80°C until analysis. For measurements of element concentrations, plasma samples were first digested with 2 mL HNO_3 for 24 hours, then treated with 30% hydrogen peroxide overnight, and finally diluted to 10 mL with deionized water. All operations were performed in a dedicated trace element clean room outfitted with a Class 100 clean hood at 20°C and 40% humidity.

2.2. ICP-MS analysis

The protocol for the ICP-MS analysis was based on previous reports [5], with minor modifications. Briefly, a total of 35 elements in plasma were analyzed using the Agilent 7850 ICP-MS system with the following parameters: fore vacuum environment, 9.5×10^{-3} mbar; analyzer pressure, 1.09×10^{-7} mbar; RF power, 1450 W; spray chamber temperature, 2°C; nebulizer gas flow, 0.90 L/min; dilution gas flow, 0.32 L/min; auxiliary gas flow, 0.8 L/min; cool gas flow 15 L/min; cell gas flow, 4 mL/min. The element concentrations (µg/L) were estimated with the 5-point standard curves with R

coefficients greater than 0.99. For each sample, the average of 3 replicate readings was reported as the element concentrations. The limit of detection (LOD) was calculated as 3 times the average of 10 consecutive measurements of the blank diluent (0.1% [v/v] TritonX-100, 1% [v/v] HNO_3 plus 10 μg/L internal standards including 6Li [No gas], 103Rh [No gas], 103Rh [He], 115In [No gas], 115In [He], 187Re [No gas], and 187Re [He]). To mitigate any potential batch effects, all samples were assayed randomly, with technicians blinded to the research data. For quality control (QC), 2 certified QC samples (ClinChek® human plasma controls for trace elements, No. 8883 and No. 8884) were inserted in each batch.

3. *Ex vivo* experiments

3.1 Isolation of PBMCs

Acid citrate dextrose tubes (BD, Franklin Lakes, NJ, USA) were used to collect venous blood from overnight fasted participants. Whole blood was centrifuged (500 *g*, 15 min) to separate the buffy-coat layer, which was further diluted in RPMI 1640 (Gibco), layered onto 3 mL of the Lymphoprep solution (1.077 g/mL density), and centrifuged (700 *g*, 30 min) to isolate PBMCs. PBMCs were washed twice in RPMI 1640, centrifuged (500 *g*, 5 min), and resuspended in extracellular flux (XF) assay buffer with 1 mM Na+ pyruvate, 1 mM GlutaMAX (Gibco), and 11 mM D-glucose. Cells were counted by the Coulter AcT diff2 Hematology Analyzer (Beckman Coulter, Brea, CA, USA) and resuspended at a density of 2.5×10^5/mL for further analyses.

3.2 Bioenergetic profiling in intact platelets

Generally, freshly isolated PBMCs were plated (250 000 cells/well) on the CellTak

-coated assay plates (BD Biosciences) in 200 μL to settle for 30 min at 37℃. The sedimented cells were attached to the bottom of the plate by centrifugation at 40 *g* and then brought up to 660 μL using XF assay medium. Cellular bioenergetics of PBMCs was determined using the Seahorse XF96 analyzer (Agilent, Santa Clara, USA).

The overall capacity of mitochondrial respiration was evaluated using the Mito Stress test, in which the oxygen consumption rates (OCRs) corresponding to basal, ATP-linked, proton leak, maximal, and reserve respiration were monitored by sequential treatment of intact platelets with oligomycin (1 μg/mL), FCCP (0.6 μM), and a mix of rotenone and antimycin A (0.5 μM each). The parameters of glycolytic rates were measured with the Glycolytic Rate assay, in which the extracellular acidification rates (ECARs, mpH/min) and OCRs (pmol/min) of PBMCs were simultaneously monitored and automatically converted into the glycolytic proton efflux rates (glycoPERs, pmol H^+/min). Then, a mix of rotenone and antimycin A (0.5 μM each) was injected to determine the maximal glycolytic capacity when mitochondrial respiration was completely blocked, followed by minimum glycoPER measurements after injection of 2-deoxy-glucose (50 mM).

The respiratory activities of individual respiratory complexes were detected by treating PBMCs with 25 μg/mL saponin first (for permeabilization), follow by injection of the following substrates: pyruvate (5 mM) + malate (2.5 mM) for complex I (CI); succinate (10 mM) for CII; duroquinol (0.5 mM) for CIII; TMPD and ascorbate (0.5/2 mM) for CIV [27]. For each respiratory complex, the rates of state 3 and state 4 respiration were estimated by sequentially monitoring OCRs on the Seahorse XF96

analyzer upon addition of ADP (1 mM) and oligomycin (1 μg/mL).

3.3 Reverse-transcription quantitative PCR (RT-qPCR)

Total RNA was extracted from PBMCs using the Trizol reagent (Sigma, cat # 93289). cDNA was synthesized with RNA to cDNA EcoDry Premix (Clontech, cat # 639547). The mRNA expression of proinflammatory genes was determined by RT-qPCR on a CFX96 Touch system (Bio-rad, Hercules, USA). RT-qPCR was performed in triplicate based on the MIQE guidelines [9]. The relative expression of a target gene was normalized to the expression of reference gene (*GAPDH*) using the $2^{-\triangle\triangle Cq}$ method [10]. The calibrator was a cDNA sample from the vehicle group. Primer sequences are as follows: 1) *IL-1β* forward: GAT GGC TTA TTA CAG TGG CA; reverse: GTA GTG GTG GTC GGA GAT T; 2) *TNF-α* forward: CCC ATG TTG TAG CAA ACC C; reverse: TGG TAG GAG ACG GCG ATG C; 3) *CXCL-10* forward: TCT CCC ATC ACT TCC CTA C; reverse: AAG AAC ATA GCA CCT CAG TA; 4) *IL-8* forward: TTG GCA GCC TTC CTG ATT T; reverse: CCT TGG GGT CCA GAC AGA G; 5) *MIP-1β* forward: TGT CTC TCC TCA TGC TAG TA; reverse: GTA CTC CTG GAC CCA GGA T; 6) *GAPDH* (reference gene) forward: GAA GGT GAA GGT CGG AGT C; reverse: GAA GAT GGT GAT GGG ATT TC.

Reference

[1] American Diabetes Association, Diagnosis and classification of diabetes mellitus, Diabetes Care. 37 Suppl 1 (2014) S81-90.

[2] Sianos G, Morel MA, Kappetein AP, Morice MC, Colombo A, Dawkins K, van den Brand M, Van Dyck N, Russell ME, Mohr FW, Serruys PW, The SYNTAX Score: an angiographic tool grading the complexity of coronary artery disease, EuroIntervention. 1 (2005) 219-227.

[3] Levine GN, Bates ER, Blankenship JC, Bailey SR, Bittl JA, Cercek B, Chambers CE, Ellis SG,

Guyton RA, Hollenberg SM, Khot UN, Lange RA, Mauri L, Mehran R, Moussa ID, Mukherjee D, Nallamothu BK, Ting HH, 2011 ACCF/AHA/SCAI Guideline for Percutaneous Coronary Intervention. A report of the American College of Cardiology Foundation/American Heart Association Task Force on Practice Guidelines and the Society for Cardiovascular Angiography and Interventions, J Am Coll Cardiol. 58 (2011) e44-122.

[4] Fihn SD, Gardin JM, Abrams J, Berra K, Blankenship JC, Dallas AP, Douglas PS, Foody JM, Gerber TC, Hinderliter AL, King SB, 3rd, Kligfield PD, Krumholz HM, Kwong RY, Lim MJ, Linderbaum JA, Mack MJ, Munger MA, Prager RL, Sabik JF, Shaw LJ, Sikkema JD, Smith CR, Jr., Smith SC, Jr., Spertus JA, Williams SV, Anderson JL, 2012 ACCF/AHA/ACP/AATS/PCNA/SCAI/STS guideline for the diagnosis and management of patients with stable ischemic heart disease: a report of the American College of Cardiology Foundation/American Heart Association task force on practice guidelines, and the American College of Physicians, American Association for Thoracic Surgery, Preventive Cardiovascular Nurses Association, Society for Cardiovascular Angiography and Interventions, and Society of Thoracic Surgeons, Circulation. 126 (2012) e354-471.

[5] Huang H, Wei L, Chen X, Zhang R, Su L, Rahman M, Golam Mostofa M, Qamruzzaman Q, Zhao Y, Yu H, Wei Y, Christiani DC, Chen F, Cord serum elementomics profiling of 56 elements depicts risk of preterm birth: Evidence from a prospective birth cohort in rural Bangladesh, Environ Int. 156 (2021) 106731.

Supplementary Table 1

Information on key reagents and commercial kits.

**Reagents**	**Source**	**Identifier**
ClinChek® Plasma Control for Trace Element	RECIPE Chemicals	Cat#: 8883 and 8884
Lymphoprep™ density gradient medium	Abcam	Cat#: ab286892
Nickel chloride	Sigma-Aldrich	Cat#: 451193
Titanium dioxide	Sigma-Aldrich	Cat#: 718467
Zirconium oxide	Sigma-Aldrich	Cat#: 544760
Oligomycin	Abcam	Cat#: 1404-19-9
FCCP	Sigma-Aldrich	Cat#: C2920
Rotenone	Sigma-Aldrich	Cat#: R8875
Antimycin A	Sigma-Aldrich	Cat#: A8674
2-Deoxy-glucose	Sigma-Aldrich	Cat#: D6134
Saponin	Sigma-Aldrich	Cat#: 558255
ADP	Sigma-Aldrich	Cat#: 01905
DHR123 probe	Invitrogen	Cat#: D23806
Sodium pyruvate	Sigma-Aldrich	Cat#: P2256
Diethyl malate	Sigma-Aldrich	Cat#: W237418
di-Sodium succinate	Sigma-Aldrich	Cat#: W327700
Duroquinol	Sigma-Aldrich	Cat#: D223204
Sodium ascorbate	Sigma-Aldrich	Cat#: PHR1279
TMPD	Sigma-Aldrich	Cat#: T7394
MitoQ mesylate	MedChemExpress	Cat#: HY-100116A
BAY 11-7082	MedChemExpress	Cat#: HY-13453
**Commercial assay**	**Source**	**Identifier**
Protein Carbonyl Content Assay Kit	Abcam	Cat#: ab126287
Lipid Peroxidation (MDA) Assay Kit	Abcam	Cat#: ab233471
8-hydroxy-2'-deoxyguanosine ELISA Kit	Abcam	Cat#: ab285254
Human Metallothionein (MT) ELISA Kit	Cusabio	Cat#: CSB-E09060h
Citrate Synthase Activity Assay Kit	Abcam	Cat#: ab119692
NADP/NADPH Assay Kit (Fluorometric)	Abcam	Cat#: ab176724
Annexin V-FITC/PI Apoptosis Detection Kit	Yeasen Biotechnology	Cat# 40302ES
MTT Assay Kit (Cell Proliferation)	Abcam	Cat#: ab211091
MILLIPLEX® Human Apolipoprotein Panel	Millipore	Product No. APOMAG-62K
MILLIPLEX® Human Adipokine Panel	Millipore	Product No. HADK1MAG-61K
Seahorse XF Cell Mito Stress Test Kit	Agilent	Part No. 103015-100
Seahorse XF Glycolytic Rate Assay Kit	Agilent	Part No. 103344-100
Amplex™ Red Assay Kit	Invitrogen	Cat#: A22188
Qproteome Mitochondria Isolation kit	Qiagen	Cat#: 37612
Pierce™ BCA Protein Assay Kits	Thermo Scientific	Cat#: 23227
TransAM® NF-κB Activation Assays	Active Motif	Cat#: 43298

Supplementary Table 2

Quality control summary of the 35 quantified elements.

Element	Abbr.	Atomic number	Type	Linearity	Intra-assay CVs (%)	Inter-assay CVs (%)	LOD (μg/L)	Detection rate (%)	Percentile		
									25th	Median	75th
Lithium	Li	7	Alkali metals	0.9997	5.85	7.52	0.016	100	2.06	4.20	6.15
Beryllium [a]	Be	9	Alkaline earth metals	0.9997	4.44	9.58	0.003	8.0	-	-	-
Boron	B	11	Metalloids	0.9998	3.50	4.29	0.080	100	20.96	30.07	38.18
Sodium	Na	23	Alkali metals	1	1.55	3.72	0.034	100	3110947	3306252	3505655
Magnesium	Mg	24	Alkaline earth metals	1	2.34	2.61	0.022	100	19449	21911	24411
Aluminum	Al	27	Post-transition metals	0.9987	3.75	5.86	0.126	100	3466	5204	6810
Potassium	K	39	Alkali metals	1	1.49	7.24	0.001	100	138831	159704	181672
Calcium	Ca	44	Alkaline earth metals	0.9998	2.02	2.92	0.001	100	84033	93016	101692
Titanium	Ti	47	Transition metals	0.9992	1.29	3.94	0.035	100	19.34	31.58	42.75
Vanadium	V	51	Transition metals	0.9997	4.80	5.87	5.6E-04	100	1.34	2.54	3.73
Chromium	Cr	52	Transition metals	0.9997	7.76	9.33	0.003	100	2.91	3.91	4.90
Manganese	Mn	55	Transition metals	0.9997	2.11	5.67	0.016	100	7.35	12.24	17.28
Iron	Fe	56	Transition metals	0.9987	2.13	9.21	0.420	100	790	1110	1458
Cobalt	Co	59	Transition metals	0.9997	2.25	7.13	8.8E-04	99.6	0.18	0.27	0.35
Nickel	Ni	60	Transition metals	0.9997	6.08	8.34	0.002	100	2.39	4.18	5.92
Copper	Cu	63	Transition metals	0.9997	2.67	7.78	0.009	100	751	1004	1243
Zinc	Zn	66	Transition metals	0.9995	5.04	9.63	0.057	100	1048	1198	1350
Gallium	Ga	71	Post-transition metals	0.9999	2.61	4.27	0.001	99.0	0.050	0.069	0.090
Arsenic	As	75	Metalloids	0.9999	7.85	7.78	0.6	97.5	1.06	1.60	2.14
Selenium	Se	78	Other nonmetals	1	2.12	8.55	0.240	100	32.28	43.82	55.64
Rubidium	Rb	85	Alkali metals	0.9999	2.00	2.16	0.004	100	122	150	178
Strontium	Sr	88	Alkaline earth metals	0.9998	6.48	7.32	0.003	100	19.49	35.28	52.32
Zirconium	Zr	90	Transition metals	0.9996	1.04	3.66	0.002	100	0.09	0.12	0.16
Niobium	Nb	93	Transition metals	0.9998	3.76	9.83	0.006	97.2	0.049	0.091	0.135
Molybdenum	Mo	95	Transition metals	1	3.09	4.97	0.006	100	1.26	1.82	2.43
Silver [a]	Ag	107	Transition metals	0.9998	3.17	6.54	2.5E-04	23.6	-	-	-
Cadmium	Cd	111	Transition metals	0.9998	1.11	8.53	6.2E-04	100	0.15	0.30	0.44
Cesium	Cs	133	Alkali metals	0.9997	1.70	2.29	3.0E-04	100	1.13	1.68	2.24
Barium	Ba	137	Alkaline earth metals	0.9998	4.22	5.05	0.010	100	16.09	29.20	42.52
Tantalum [a]	Ta	181	Transition metals	1	4.77	8.24	0.003	7.0	-	-	-
Tungsten [a]	W	182	Transition metals	1	2.24	5.83	0.005	38.0	-	-	-
Rhenium [a]	Re	185	Transition metals	0.9999	3.53	4.03	1.5E-04	25.3	-	-	-
Thallium	Tl	205	Post-transition metals	0.9998	3.76	5.28	0.002	97.8	0.07	0.12	0.16
Lead	Pb	208	Post-transition metals	0.9998	5.54	8.76	0.007	100	7.10	14.00	20.33
Uranium	U	238	Actinoids	0.9998	3.57	5.59	1.7E-04	100	0.048	0.065	0.082

[a] Elements with low detection rates (<50%) were categorized as “detectable” and “undetectable”.

Supplementary Table 3

Associations of 35 plasma elements with the global oxidative stress marker ORP.

Elements	N	cORP				sORP			
		Model 1 [a]		Model 2 [b]		Model 1 [a]		Model 2 [b]	
		Percent change	*P* value	Percent change	*P* value	Percent change	*P* value/FDR	Percent change	*P* value/FDR
Nickel									
Q1	786	Reference		Reference		Reference		Reference	
Q2	785	-17.2 (-39.5, 13.4)		-17.7 (-39.9, 12.7)		25.8 (4.9, 50.9)		23.2 (3.2, 48.4)	
Q3	785	-34.6 (-52.2, -10.4)		-35.3 (-52.7, -11.3)		22.1 (1.8, 46.4)		20.3 (0.4, 44.3)	
Q4	786	-33.0 (-51.1, -8.3)		-32.9 (-51.1, -8.1)		41.0 (17.1, 69.1)		38.2 (15.2, 65.8)	
Continuous	3142	-15.9 (-26.8, -3.3)	0.015/0.53	-16.1 (-27.0, -3.5)	0.014/0.39	14.9 (6.1, 24.5)	**<0.001**	14.2 (5.4, 23.8)	**0.001/0.035**
Zirconium									
Q1	785	Reference		Reference		Reference		Reference	
Q2	790	16.0 (-15.3, 58.8)		18.1 (-13.8, 61.8)		7.6 (-10.3, 29.0)		7.2 (-10.6, 28.6)	
Q3	783	2.9 (-24.9, 41.0)		3.3 (-24.6, 41.6)		12.8 (-6.0, 35.3)		12.4 (-6.3, 34.8)	
Q4	784	-8.2 (-33.0, 25.8)		-7.8 (-32.7, 26.3)		28.9 (7.5, 54.6)		27.9 (6.6, 53.4)	
Continuous	3142	-17.7 (-38.5, 10.2)	0.19	-17.4 (-38.3, 10.5)	0.20	31.5 (11.1, 55.6)	**0.001**	30.7 (10.5, 54.7)	**0.002/0.035**
Titanium									
Q1	785	Reference		Reference		Reference		Reference	
Q2	786	1.8 (-25.7, 39.4)		2.6 (-25.1, 40.6)		3.9 (-13.4, 24.6)		4.0 (-13.3, 24.7)	
Q3	787	-6.8 (-31.9, 27.7)		-7.1 (-32.2, 27.3)		13.9 (-5.1, 36.6)		13.8 (-5.1, 36.4)	
Q4	784	-11.6 (-35.5, 21.2)		-10.6 (-34.8, 22.5)		32.2 (10.2, 58.6)		31.8 (9.9, 58.1)	
Continuous	3142	-11.9 (-28.3, 8.4)	0.23	-11.6 (-28.1, 8.7)	0.24	20.3 (6.7, 35.5)	**0.002**	20.1 (6.6, 35.3)	**0.003/0.035**
Strontium									
Q1	790	Reference		Reference		Reference		Reference	
Q2	783	10.0 (-19.7, 50.7)		9.0 (-20.5, 49.3)		16.4 (-2.9, 39.7)		14.5 (-4.5, 37.4)	
Q3	784	4.9 (-23.3, 43.7)		4.7 (-23.5, 43.4)		7.5 (-10.3, 28.9)		7.5 (-10.3, 28.9)	
Q4	785	-10.7 (-34.7, 22.3)		-10.9 (-34.9, 22.0)		22.2 (1.9, 46.6)		21.2 (1.1, 45.3)	
Continuous	3142	-11.0 (-23.3, 3.3)	0.12	-11.0 (-23.3, 3.2)	0.12	10.5 (1.4, 20.4)	0.023	10.3 (1.2, 20.2)	0.025/0.22
Vanadium									
Q1	785	Reference		Reference		Reference		Reference	
Q2	786	-12.1 (-35.8, 20.4)		-12.5 (-36.1, 19.8)		5.2 (-12.3, 26.1)		4.1 (-13.1, 24.9)	
Q3	787	-5.0 (-30.7, 30.0)		-6.1 (-31.4, 28.6)		10.8 (-7.6, 32.9)		10.7 (-7.7, 32.7)	
Q4	784	-10.3 (-34.5, 22.8)		-10.2 (-34.5, 23.1)		15.3 (-3.9, 38.3)		13.3 (-5.6, 35.9)	
Continuous	3142	-2.6 (-15.8, 12.8)	0.73	-2.8 (-16.1, 12.6)	0.70	8.3 (-0.5, 17.9)	0.07	7.7 (-1.1, 17.2)	0.09
Barium									
Q1	785	Reference		Reference		Reference		Reference	
Q2	786	3.3 (-24.5, 41.5)		4.1 (-24.0, 42.6)		-3.8 (-19.8, 15.3)		-4.2 (-20.1, 14.9)	
Q3	787	-4.5 (-30.2, 30.8)		-3.9 (-29.9, 31.6)		12.8 (-5.9, 35.3)		11.9 (-6.7, 34.2)	
Q4	784	27.3 (-7.1, 74.4)		27.9 (-6.7, 75.2)		11.4 (-7.2, 33.6)		10.1 (-8.3, 32.0)	
Continuous	3142	9.8 (-5.6, 27.8)	0.22	10.2 (-5.3, 28.2)	0.21	6.4 (-2.5, 16.2)	0.16	5.7 (-3.1, 15.4)	0.21

Selenium									
Q1	787	Reference		Reference		Reference		Reference	
Q2	784	1.6 (-25.9, 39.2)		0.2 (-26.9, 37.4)		11.3 (-7.2, 33.6)		12.5 (-6.2, 35.0)	
Q3	787	23.7 (-9.6, 69.4)		23.2 (-10.1, 68.8)		-5.1 (-20.9, 13.9)		-4.8 (-20.6, 14.2)	
Q4	784	4.8 (-23.5, 43.6)		3.4 (-24.5, 41.8)		-6.9 (-22.4, 11.7)		-7.1 (-22.6, 11.4)	
Continuous	3142	4.4 (-25.1, 45.6)	0.80	4.1 (-25.4, 45.2)	0.81	-10.5 (-26.2, 8.5)	0.26	-10.9 (-26.5, 8.0)	0.24
Boron									
Q1	785	Reference		Reference		Reference		Reference	
Q2	785	-14.7 (-37.7, 16.8)		-15.8 (-38.5, 15.3)		3.4 (-13.8, 24.1)		3.2 (-14.0, 23.8)	
Q3	787	15.9 (-15.4, 58.7)		15.8 (-15.5, 58.6)		-2.3 (-18.6, 17.2)		-2.9 (-19.0, 16.5)	
Q4	785	-8.0 (-32.8, 25.9)		-9.2 (-33.7, 24.3)		18.7 (-1.0, 42.4)		19.5 (-0.4, 43.3)	
Continuous	3142	-2.6 (-28.6, 32.8)	0.87	-3.6 (-29.3, 31.6)	0.82	11.0 (-7.3, 32.8)	0.26	11.2 (-7.1, 33.0)	0.25
Iron									
Q1	786	Reference		Reference		Reference		Reference	
Q2	788	-0.9 (-27.6, 35.7)		-1.6 (-28.2, 34.8)		-8.0 (-23.3, 10.4)		-8.0 (-23.3, 10.3)	
Q3	784	4.1 (-24.0, 42.7)		5.1 (-23.4, 44.0)		4.9 (-12.6, 25.9)		4.0 (-13.0, 24.8)	
Q4	784	20.3 (-12.2, 64.8)		19.8 (-12.6, 64.1)		-11.9 (-26.6, 5.7)		-12.6 (-27.1, 4.8)	
Continuous	3142	-14.6 (-14.9, 54.1)	0.37	14.7 (-14.8, 54.4)	0.37	-8.7 (-23.1, 8.4)	0.30	-9.4 (-23.7, 7.6)	0.26
Aluminum									
Q1	785	Reference		Reference		Reference		Reference	
Q2	786	-10.7 (-34.8, 22.3)		-12.7 (-36.3, 19.6)		0.3 (-16.4, 20.3)		0.8 (-15.9, 20.9)	
Q3	786	-9.2 (-33.7, 24.4)		-9.2 (-33.7, 24.4)		-2.0 (-18.3, 17.5)		-1.6 (-17.9, 18.1)	
Q4	785	14.7 (-16.3, 57.0)		15.5 (-15.7, 58.2)		12.6 (-6.2, 35.0)		13.8 (-5.1, 36.5)	
Continuous	3142	12.0 (-8.5, 37.1)	0.27	12.8 (-7.8, 38.1)	0.24	6.2 (-5.5, 19.3)	0.32	6.7 (-5.1, 19.9)	0.28
Cobalt									
Q1	788	Reference		Reference		Reference		Reference	
Q2	783	23.5 (-9.8, 69.2)		22.5 (-10.6, 67.8)		9.3 (-8.9, 31.1)		9.3 (-8.9, 31.1)	
Q3	787	16.5 (-14.9, 59.5)		16.2 (-15.2, 59.1)		12.3 (-6.3, 34.7)		11.2 (-7.1, 33.4)	
Q4	784	30.0 (-5.1, 78.1)		27.9 (-6.6, 75.3)		12.2 (-6.5, 34.6)		12.7 (-6.0, 35.2)	
Continuous	3142	10.0 (-9.0, 32.9)	0.33	9.5 (-9.4, 32.4)	0.35	5.4 (-5.6, 17.6)	0.35	6.1 (-4.9, 18.4)	0.29
Potassium									
Q1	785	Reference		Reference		Reference		Reference	
Q2	786	-21.8 (-42.9, 7.1)		-21.0 (-42.4, 8.2)		6.7 (-11.1, 28.0)		6.4 (-11.3, 27.6)	
Q3	786	-22.7 (-43.5, 5.9)		-22.8 (-43.7, 5.7)		14.8 (-4.3, 37.7)		15.8 (-3.4, 38.9)	
Q4	785	-17.3 (-39.6, 13.2)		-16.3 (-38.9, 14.6)		8.9 (-9.3, 30.6)		9.6 (-8.6, 31.5)	
Continuous	3142	-3.9 (-7.0, 2.4)	0.17	-3.9 (-7.0, 2.5)	0.18	2.2 (-1.9, 8.4)	0.35	2.2 (-1.9, 6.5)	0.29
Lead									
Q1	785	Reference		Reference		Reference		Reference	
Q2	786	1.7 (-25.7, 39.3)		0.5 (-26.6, 37.7)		5.1 (-12.4, 26.1)		5.4 (-12.1, 26.4)	
Q3	787	-11.0 (-35.0, 21.9)		-11.1 (-35.1, 21.7)		19.7 (-0.2, 43.6)		19.8 (-0.1, 43.7)	
Q4	784	10.7 (-19.2, 51.7)		12.4 (-18.0, 54.0)		12.4 (-6.3, 34.9)		12.9 (-5.9, 35.4)	
Continuous	3142	3.4 (-10.2, 19.0)	0.64	3.8 (-9.8, 19.5)	0.60	4.3 (-3.9, 13.1)	0.31	4.4 (-3.8, 12.2)	0.30

Copper									
Q1	785	Reference		Reference		Reference		Reference	
Q2	787	33.3 (-2.6, 82.6)		33.4 (-2.6, 82.7)		1.9 (-15.1, 22.2)		2.5 (-14.5, 22.9)	
Q3	786	30.8 (-4.5, 79.1)		32.0 (-3.6, 80.7)		11.5 (-7.1, 33.7)		12.3 (-6.4, 34.6)	
Q4	784	14.7 (-16.3, 57.1)		16.3 (-15.1, 59.3)		4.3 (-13.1, 25.1)		4.2 (-13.1, 25.0)	
Continuous	3142	16.1 (-19.0, 66.4)	0.42	17.8 (-17.8, 69.0)	0.37	9.1 (-11.4, 34.4)	0.41	9.2 (-11.3, 34.5)	0.41
Thallium									
Q1	794	Reference		Reference		Reference		Reference	
Q2	779	25.0 (-8.7, 71.2)		25.1 (-8.6, 71.3)		7.6 (-10.3, 29.0)		7.0 (-10.8, 28.3)	
Q3	785	1.8 (-25.6, 39.3)		2.0 (-25.5, 39.6)		19.1 (-0.7, 42.8)		16.9 (-2.5, 40.1)	
Q4	784	13.0 (-17.4, 54.6)		13.2 (-17.3, 55.0)		16.7 (-2.6, 40.0)		16.3 (-3.0, 39.4)	
Continuous	3142	4.0 (-9.0, 19.0)	0.56	3.7 (-9.4, 18.6)	0.60	3.5 (-4.3, 11.8)	0.39	3.3 (-4.4, 11.7)	0.41
Manganese									
Q1	785	Reference		Reference		Reference		Reference	
Q2	786	-7.9 (-32.7, 26.2)		-6.6 (-31.8, 28.0)		11.1 (-7.4, 33.3)		11.7 (-6.9, 33.9)	
Q3	788	-5.8 (-31.2, 29.0)		-5.7 (-31.2, 29.1)		0.9 (-15.9, 21.0)		1.6 (-15.2, 21.9)	
Q4	783	11.4 (-18.7, 52.7)		11.5 (-18.7, 52.7)		-8.2 (-23.5, 10.2)		-8.2 (-23.4, 10.1)	
Continuous	3142	-7.9 (-11.1, 31.0)	0.44	8.0 (-11.0, 31.1)	0.44	-4.5 (-14.6, 6.9)	0.42	-4.3 (-14.4, 7.1)	0.44
Chromium									
Q1	788	Reference		Reference		Reference		Reference	
Q2	791	-4.0 (-29.8, 31.4)		-3.2 (-29.2, 32.5)		-7.8 (-23.1, 10.5)		-8.1 (-23.4, 10.1)	
Q3	784	-21.8 (-42.9, 7.2)		-21.3 (-42.5, 7.9)		-4.3 (-20.3, 14.8)		-4.3 (-20.3, 14.8)	
Q4	779	-9.0 (-33.6, 24.7)		-8.0 (-32.9, 26.1)		-6.9 (-22.4, 11.8)		-6.2 (-21.8, 12.5)	
Continuous	3142	-11.2 (-37.3, 25.8)	0.51	-10.2 (-36.6, 27.3)	0.55	-6.9 (-23.9, 13.9)	0.49	-6.5 (-23.5, 14.3)	0.51
Sodium									
Q1	785	Reference		Reference		Reference		Reference	
Q2	786	-18.7 (-40.6, 11.3)		-20.8 (-42.2, 8.5)		-0.2 (-16.8, 19.7)		0.6 (-16.1, 20.7)	
Q3	786	-10.8 (-34.9, 22.2)		-11.0 (-35.0, 22.0)		-0.2 (-16.8, 19.8)		-0.3 (-16.9, 19.6)	
Q4	785	-13.4 (-36.7, 18.7)		-13.8 (-37.1, 18.1)		-5.1 (-20.9, 13.9)		-4.5 (-20.4, 14.5)	
Continuous	3142	-5.6 (-9.1, 11.9)	0.32	-5.5 (-9.1, 12.3)	0.33	-2.7 (-7.1, 8.3)	0.50	-3.0 (-11.6, 6.5)	0.52
Lithium									
Q1	788	Reference		Reference		Reference		Reference	
Q2	783	10.4 (-19.4, 51.1)		9.7 (-19.9, 50.3)		11.4 (-7.1, 33.7)		10.7 (-7.7, 32.8)	
Q3	789	22.9 (-10.2, 68.1)		22.3 (-10.7, 67.4)		4.5 (-12.8, 25.3)		5.1 (-12.4, 26.0)	
Q4	782	12.8 (-17.6, 54.6)		13.3 (-17.3, 55.2)		13.9 (-5.0, 36.7)		13.9 (-5.0, 36.6)	
Continuous	3142	9.2 (-2.9, 22.9)	0.14	9.1 (-3.1, 22.8)	0.15	2.0 (-4.7, 9.2)	0.57	1.8 (-4.9, 9.0)	0.61
Zinc									
Q1	787	Reference		Reference		Reference		Reference	
Q2	787	-9.2 (-33.7, 24.3)		-9.7 (-34.1, 23.7)		16.5 (-2.9, 39.7)		16.4 (-3.0, 39.5)	
Q3	784	-11.4 (-35.3, 21.3)		-11.8 (-35.6, 20.9)		11.0 (-7.4, 25.5)		12.6 (-6.1, 35.0)	
Q4	784	-14.7 (-37.7, 16.9)		-15.6 (-38.3, 15.7)		4.6 (-12.8, 25.5)		5.3 (-12.2, 26.3)	
Continuous	3142	-3.4 (-6.9, 4.1)	0.28	-3.5 (-7.0, 3.9)	0.27	0.9 (-3.0, 6.9)	0.70	1.1 (-3.2, 5.7)	0.62

Uranium									
Q1	781	Reference		Reference		Reference		Reference	
Q2	791	-10.4 (-34.6, 22.8)		-10.3 (-34.6, 22.9)		-4.3 (-20.2, 14.9)		-3.8 (-19.8, 15.4)	
Q3	787	-8.9 (-33.5, 24.8)		-9.9 (-34.3, 23.4)		-9.8 (-24.8, 8.2)		-9.6 (-24.6, 8.5)	
Q4	783	-18.5 (-40.5, 11.7)		-19.1 (-41.0, 10.9)		-0.4 (-17.0, 19.6)		-1.1 (-17.6, 18.7)	
Continuous	3142	-18.9 (-42.2, 13.8)	0.23	-19.7 (-42.8, 12.8)	0.21	-4.1 (-21.2, 16.7)	0.67	-4.8 (-21.8, 15.8)	0.62
Magnesium									
Q1	785	Reference		Reference		Reference		Reference	
Q2	786	17.3 (-14.4, 60.6)		16.0 (-15.4, 59.0)		9.3 (-8.9, 31.1)		9.1 (-9.0, 30.9)	
Q3	786	2.0 (-25.5, 39.7)		1.0 (-26.3, 38.5)		5.5 (-12.1, 26.6)		6.1 (-11.5, 27.3)	
Q4	785	2.8 (-24.9, 40.8)		1.4 (-26.0, 39.0)		4.6 (-12.8, 25.5)		4.4 (-13.0, 25.2)	
Continuous	3142	-0.1 (-5.7, 12.9)	0.98	-0.4 (-5.9, 12.1)	0.92	1.2 (-3.1, 8.2)	0.65	1.2 (-3.6, 6.2)	0.64
Molybdenum									
Q1	788	Reference		Reference		Reference		Reference	
Q2	783	24.8 (-8.8, 70.8)		23.0 (-10.2, 68.5)		-4.2 (-20.2, 14.9)		-4.1 (-20.1, 15.0)	
Q3	786	2.6 (-25.0, 40.5)		2.7 (-25.0, 40.5)		-7.4 (-22.8, 11.1)		-8.0 (-23.3, 10.3)	
Q4	785	47.8 (8.0, 102.4)		48.3 (8.3, 103.1)		-5.8 (-21.5, 13.0)		-6.6 (-22.1, 12.0)	
Continuous	3142	27.2 (-3.7, 68.0)	0.09	28.0 (-3.1, 69.1)	0.08	-2.8 (-17.3, 14.2)	0.73	-3.5 (-17.8, 13.4)	0.67
Niobium									
Q1	789	Reference		Reference		Reference		Reference	
Q2	801	3.6 (-24.2, 41.6)		2.8 (-24.8, 40.6)		-2.5 (-18.6, 16.8)		-1.1 (-17.4, 18.5)	
Q3	777	4.0 (-24.1, 42.5)		4.1 (-24.1, 42.7)		-15.6 (-29.7, 1.3)		-14.2 (-28.5, 2.9)	
Q4	775	-9.2 (-33.8, 24.4)		-9.1 (-33.7, 24.7)		-7.1 (-22.6, 11.5)		-5.5 (-21.2, 13.4)	
Continuous	3142	-4.0 (-15.3, 8.8)	0.52	-4.1 (-15.4, 8.7)	0.52	-2.1 (-8.9, 5.3)	0.57	-1.5 (-8.4, 5.9)	0.68
Cadmium									
Q1	785	Reference		Reference		Reference		Reference	
Q2	786	-8.8 (-33.4, 24.8)		-9.5 (-34.0, 24.0)		-3.4 (-19.5, 15.8)		-3.2 (-19.3, 16.2)	
Q3	786	-24.0 (-44.5, 4.1)		-23.8 (-44.4, 4.3)		-7.1 (-22.5, 11.5)		-7.2 (-22.6, 11.3)	
Q4	785	-13.9 (-37.2, 17.9)		-14.4 (-37.5, 17.3)		-12.4 (-27.0, 5.1)		-12.0 (-26.7, 5.5)	
Continuous	3142	-9.1 (-18.9, 2.0)	0.10	-8.9 (-18.8, 2.1)	0.11	-1.5 (-7.8, 5.3)	0.66	-1.4 (-7.7, 5.4)	0.69
Rubidium									
Q1	786	Reference		Reference		Reference		Reference	
Q2	786	-20.0 (-41.6, 9.5)		-20.2 (-41.7, 9.3)		3.3 (-13.9, 23.9)		3.2 (-13.9, 23.8)	
Q3	788	0.4 (-26.6, 37.5)		-0.1 (-27.1, 36.7)		0.6 (-16.1, 20.6)		0.9 (-15.9, 21.0)	
Q4	782	-24.3 (-44.7, 3.7)		-23.9 (-44.4, 4.2)		4.8 (-12.7, 25.7)		4.3 (-13.0, 25.1)	
Continuous	3142	-33.8 (-59.6, 8.6)	0.10	-33.4 (-59.4, 9.2)	0.11	6.2 (-20.3, 41.4)	0.68	5.7 (-20.6, 40.7)	0.71
Gallium									
Q1	786	Reference		Reference		Reference		Reference	
Q2	787	10.7 (-19.2, 51.5)		10.9 (-19.0, 51.9)		10.5 (-7.8, 32.6)		11.5 (-7.1, 33.7)	
Q3	784	27.8 (-6.7, 75.0)		26.2 (-7.9, 72.9)		19.9 (-0.1, 43.8)		21.3 (1.2, 45.5)	
Q4	785	-2.5 (-28.8, 33.5)		-2.3 (-28.7, 33.8)		3.8 (-13.5, 24.5)		4.4 (-13.0, 25.2)	
Continuous	3142	10.6 (-8.1, 33.0)	0.29	11.1 (-7.6, 33.6)	0.26	-1.7 (-11.6, 9.4)	0.76	-2.3 (-15.7, 13.3)	0.84

Cesium									
Q1	798	Reference		Reference		Reference		Reference	
Q2	776	26.8 (-7.4, 73.6)		26.1 (-7.9, 72.8)		9.5 (-8.7, 31.3)		9.6 (-8.6, 31.5)	
Q3	793	2.2 (-25.3, 39.8)		1.5 (-25.8, 38.9)		-5.2 (-20.9, 13.6)		-4.2 (-20.1, 14.8)	
Q4	775	9.0 (-20.4, 49.2)		6.9 (-22.0, 46.5)		1.6 (-15.3, 21.9)		1.4 (-15.5, 21.6)	
Continuous	3142	8.3 (-16.3, 40.1)	0.61	6.7 (-17.6, 38.1)	0.62	0.2 (-13.6, 16.3)	0.98	0.5 (-13.5, 16.6)	0.95
Calcium									
Q1	785	Reference		Reference		Reference		Reference	
Q2	786	-2.3 (-28.6, 33.9)		-2.4 (-28.8, 33.7)		23.1 (2.6, 47.6)		23.6 (3.0, 48.2)	
Q3	786	4.3 (-23.8, 42.9)		4.6 (-23.6, 43.4)		3.7 (-13.6, 24.4)		3.7 (-13.6, 24.3)	
Q4	785	0.5 (-26.7, 37.6)		0.2 (-26.9, 37.3)		4.7 (-12.7, 25.6)		4.7 (-12.8, 25.5)	
Continuous	3142	-0.3 (-6.5, 17.3)	0.96	-0.5 (-6.6, 16.8)	0.93	-0.2 (-4.6, 7.8)	0.94	-1.5 (-5.9, 6.0)	0.96
Arsenic									
Q1	784	Reference		Reference		Reference		Reference	
Q2	787	3.2 (-24.7, 41.4)		3.8 (-24.2, 42.2)		16.9 (-2.6, 40.2)		16.2 (-3.1, 39.4)	
Q3	787	12.2 (-18.1, 53.7)		12.1 (-18.1, 53.6)		-0.9 (-17.4, 18.9)		-0.6 (-17.1, 19.2)	
Q4	784	-4.5 (-30.3, 30.9)		-3.9 (-29.9, 31.6)		7.3 (-10.6, 28.7)		6.3 (-11.4, 27.5)	
Continuous	3142	-2.1 (-24.3, 26.8)	0.87	-1.7 (-24.0, 27.3)	0.90	0.9 (-13.1, 17.1)	0.91	0.3 (-13.6, 16.4)	0.97
Silver									
Undetectable	2399	Reference		Reference		Reference		Reference	
Detectable	743	-10.4 (-31.0, 16.4)	0.41	-9.8 (-30.6, 17.2)	0.44	5.1 (-9.7, 22.3)	0.52	5.9 (-9.0, 23.2)	0.46
Tantalum									
Undetectable	2923	Reference		Reference		Reference		Reference	
Detectable	219	17.1 (-24.3, 81.2)	0.48	19.5 (-22.8, 85.0)	0.42	8.6 (-15.7, 39.8)	0.52	8.6 (-15.6, 39.7)	0.52
Tungsten									
Undetectable	1948	Reference		Reference		Reference		Reference	
Detectable	1194	19.3 (-5.1, 50.0)	0.13	18.2 (-6.0, 48.7)	0.15	2.8 (-9.9, 17.4)	0.68	3.7 (-9.1, 18.4)	0.59
Rhenium									
Undetectable	2346	Reference		Reference		Reference		Reference	
Detectable	796	6.6 (-17.4, 37.7)	0.62	8.8 (-15.8, 40.5)	0.52	-2.7 (-16.1, 12.8)	0.72	-2.3 (-15.7, 13.3)	0.76
Beryllium									
Undetectable	2891	Reference		Reference		Reference		Reference	
Detectable	251	-25.8 (-50.8, 11.8)	0.15	-26.9 (-51.5, 10.1)	0.13	3.0 (-18.8, 30.6)	0.81	3.0 (-18.7, 30.6)	0.81

[a] Model 1 was adjusted for age, sex, body mass index, cigarette smoking, and alcohol drinking.

[b] Model 2 was adjusted for age, sex, body mass index, cigarette smoking, alcohol drinking, creatinine clearance, C-reactive protein, hypertension, dyslipidemia, peripheral vascular disease, chronic obstructive pulmonary disease, glycated hemoglobin, fasting glucose, diabetes duration, and management of diabetes.

Supplementary Table 4

Sensitivity analyses for estimating percent changes in sORP when simultaneously including nickel, zirconium, and titanium in the regression model.

		Model 1 [a]		Model 2 [b]	
Elements	N	Percent change	*P* value	Percent change	*P* value
Nickel					
Q1	786	Reference		Reference	
Q2	785	25.9 (5.0, 50.9)		23.9 (3.3, 48.5)	
Q3	785	22.7 (2.4, 47.1)		20.9 (0.9, 44.9)	
Q4	786	41.0 (17.6, 69.1)		38.3 (15.4, 65.9)	
Continuous	3142	15.1 (6.3, 24.7)	5.7E-4	14.4 (5.6, 23.9)	0.001
Zirconium					
Q1	785	Reference		Reference	
Q2	790	7.5 (-10.3, 28.8)		7.2 (-10.6, 28.5)	
Q3	783	13.2 (-5.6, 35.7)		12.9 (-5.9, 35.3)	
Q4	784	29.0 (7.6, 54.7)		28.0 (6.8, 53.5)	
Continuous	3142	31.8 (11.5, 55.9)	0.001	31.0 (10.8, 55.0)	0.002
Titanium					
Q1	785	Reference		Reference	
Q2	786	4.9 (-12.5, 25.8)		5.0 (-12.4, 25.8)	
Q3	787	15.3 (-3.8, 38.2)		15.2 (-3.9, 38.1)	
Q4	784	32.6 (10.6, 59.0)		32.2 (10.3, 58.5)	
Continuous	3142	20.7 (7.2, 36.0)	0.002	20.5 (7.0, 35.7)	0.002

[a] Model 1 was adjusted for age, sex, body mass index, cigarette smoking, and alcohol drinking.

[b] Model 2 was adjusted for age, sex, body mass index, cigarette smoking, alcohol drinking, creatinine clearance, C-reactive protein, hypertension, dyslipidemia, peripheral vascular disease, chronic obstructive pulmonary disease, glycated hemoglobin, fasting glucose, diabetes duration, and management of diabetes.

Supplementary Table 5

Associations of each 1-quartile increase in ORP values and element concentrations with obstructive CAD and CAD index.

Variables	Obstructuve CAD			CAD index		
	OR (95%CI) [b]	*P* value [b]	FDR	OR (95%CI) [b]	*P* value [b]	FDR
**ORP**						
sORP	1.20 (1.12, 1.28)	<0.001	**<0.001**	1.13 (1.07, 1.20)	<0.001	**<0.001**
cORP	0.83 (0.78, 0.89)	<0.001	**<0.001**	0.88 (0.83, 0.93)	<0.001	**<0.001**
**Elements**						
Zirconium	1.22 (1.14, 1.30)	<0.001	**<0.001**	1.15 (1.09, 1.22)	<0.001	**<0.001**
Strontium	1.22 (1.14, 1.30)	<0.001	**<0.001**	1.16 (1.10, 1.23)	<0.001	**<0.001**
Vanadium	1.19 (1.11, 1.27)	<0.001	**<0.001**	1.15 (1.09, 1.21)	<0.001	**<0.001**
Titanium	1.13 (1.06, 1.21)	<0.001	**0.001**	1.08 (1.02, 1.14)	0.008	**0.042**
Nickel	1.11 (1.04, 1.18)	0.002	**0.011**	1.08 (1.03, 1.15)	0.004	**0.025**
Aluminum	1.09 (1.02, 1.16)	0.012	0.06	1.05 (0.99, 1.11)	0.10	0.28
Rhenium [a]	1.17 (0.99, 1.39)	0.06	0.25	1.15 (0.99, 1.32)	0.06	0.25
Beryllium [a]	1.24 (0.95, 1.62)	0.12	0.44	1.29 (1.03, 1.62)	0.029	0.13
Gallium	0.96 (0.90, 1.02)	0.19	0.54	0.99 (0.93, 1.04)	0.60	0.94
Silver [a]	1.12 (0.95, 1.33)	0.19	0.54	1.05 (0.91, 1.21)	0.50	0.88
Tungsten [a]	1.10 (0.95, 1.28)	0.19	0.54	1.12 (0.99, 1.27)	0.08	0.27
Selenium	0.96 (0.90, 1.03)	0.26	0.68	0.96 (0.91, 1.02)	0.17	0.45
Cobalt	0.97 (0.91, 1.03)	0.34	0.68	0.96 (0.91, 1.02)	0.20	0.49
Potassium	1.03 (0.97, 1.10)	0.31	0.68	1.03 (0.97, 1.09)	0.33	0.64
Uranium	1.03 (0.97, 1.10)	0.35	0.68	1.01 (0.96, 1.07)	0.71	0.94
Niobium	1.03 (0.97, 1.10)	0.35	0.68	0.99 (0.94, 1.05)	0.83	0.94
Cesium	0.97 (0.91, 1.03)	0.33	0.68	0.97 (0.91, 1.02)	0.22	0.50
Copper	0.97 (0.91, 1.04)	0.39	0.72	0.95 (0.90, 1.01)	0.08	0.27
Sodium	0.97 (0.91, 1.04)	0.41	0.72	0.99 (0.93, 1.04)	0.66	0.94
Thallium	1.03 (0.96, 1.09)	0.45	0.76	0.99 (0.94, 1.05)	0.79	0.94
Barium	1.02 (0.96, 1.09)	0.51	0.80	0.99 (0.93, 1.04)	0.66	0.94
Tantalum [a]	1.10 (0.83, 1.46)	0.52	0.80	1.00 (0.79, 1.26)	0.99	0.99
Zinc	1.02 (0.96, 1.09)	0.57	0.81	1.05 (0.99, 1.11)	0.10	0.28
Arsenic	1.02 (0.95, 1.09)	0.57	0.81	1.01 (0.96, 1.07)	0.66	0.94
Boron	0.99 (0.92, 1.05)	0.66	0.82	0.97 (0.92, 1.03)	0.33	0.64
Chromium	1.01 (0.95, 1.08)	0.69	0.82	0.99 (0.94, 1.05)	0.73	0.94
Lithium	1.02 (0.95, 1.08)	0.65	0.82	1.01 (0.95, 1.06)	0.84	0.94
Molybdenum	1.01 (0.95, 1.08)	0.67	0.82	1.03 (0.98, 1.09)	0.23	0.50
Calcium	0.98 (0.92, 1.05)	0.64	0.82	0.99 (0.94, 1.05)	0.83	0.94
Magnesium	0.99 (0.93, 1.05)	0.72	0.83	1.03 (0.97, 1.08)	0.36	0.67
Cadmium	0.99 (0.93, 1.06)	0.83	0.93	0.99 (0.94, 1.05)	0.77	0.94
Iron	1.00 (0.94, 1.07)	0.96	0.99	1.00 (0.94, 1.06)	0.95	0.99
Lead	1.00 (0.94, 1.07)	0.95	0.99	1.00 (0.95, 1.06)	0.97	0.99
Manganese	1.00 (0.94, 1.07)	0.97	0.99	1.00 (0.95, 1.06)	0.90	0.98
Rubidium	1.00 (0.94, 1.07)	0.99	0.99	0.99 (0.93, 1.04)	0.63	0.94

[a] For elements with low detection rates, effects estimates were calculated by comparing the detectable group to the undetectable group.

[b] Adjusted for age, sex, body mass index, cigarette smoking, alcohol drinking, creatinine clearance, C-reactive protein, hypertension, dyslipidemia, peripheral vascular disease, chronic obstructive pulmonary disease, glycated hemoglobin, fasting glucose, diabetes duration, and management of diabetes.

Supplementary Table 6

Comparisons of median concentration of 5 redox-related metals among nondiabetic subjects, type 2 diabetic patients without obstructive CAD, and type 2 diabetic patients with obstructive CAD.

Redox-related metals	Nondiabetic subjects		T2DM without obstructive CAD		T2DM with obstructive CAD		P_{trend} [b]
	N	Median (IQR)	N	Median (IQR)	N	Median (IQR)	
Zirconium, μg/L [a]	1000	-2.32 (-2.77, -1.93)	1672	-2.15 (-2.48, -1.88)	1470	-2.05 (-2.41, -1.77)	<0.001
Titanium, μg/L [a]	1000	3.33 (2.81, 3.69)	1672	3.42 (2.92, 3.72)	1470	3.50 (3.01, 3.80)	<0.001
Nickel, μg/L [a]	1000	1.32 (0.61, 1.72)	1672	1.38 (0.83, 1.75)	1470	1.49 (0.94, 1.82)	<0.001
Strontium, μg/L [a]	1000	3.41 (2.66, 3.84)	1672	3.49 (2.89, 3.89)	1470	3.66 (3.02, 4.05)	<0.001
Vanadium, μg/L [a]	1000	0.74 (0.12, 1.15)	1672	0.86 (0.24, 1.25)	1470	1.02 (0.35, 1.41)	<0.001
5-metal mixture	1000	0.23 (0.13, 0.34)	1672	0.31 (0.23, 0.38)	1470	0.35 (0.27, 0.43)	<0.001

[a] Metal concentrations were ln-transformed before analysis.

[b] Calculated from the ordinal logistic regression model with adjustment for age, sex, body mass index, cigarette smoking, alcohol drinking, hypertension, dyslipidemia, peripheral vascular disease, and chronic obstructive pulmonary disease.

Supplementary Table 7

Prospective associations of each 1-quartile increase in ORP values and element concentrations with 2-year MACCE after PCI

Variable	HR (95%CI)	*P* value	FDR
**ORP**			
sORP	1.33 (1.19-1.50)	**<0.001**	**<0.001**
cORP	0.89 (0.79-1.00)	0.06	0.28
**Elements**			
Zirconium	1.21 (1.08-1.36)	**0.001**	**0.016**
Strontium	1.00 (0.89-1.12)	0.98	0.99
Vanadium	0.99 (0.89-1.12)	0.92	0.99
Titanium	1.21 (1.07-1.36)	**0.002**	**0.016**
Nickel	1.25 (1.11-1.40)	**<0.001**	**0.004**
Aluminum	0.94 (0.84-1.06)	0.31	0.78
Rhenium [a]	1.08 (0.81-1.44)	0.60	0.88
Beryllium [a]	1.05 (0.67-1.65)	0.84	0.99
Gallium	1.12 (1.00-1.25)	0.06	0.28
Silver [a]	1.12 (0.84-1.51)	0.44	0.81
Tungsten [a]	0.97 (0.75-1.27)	0.84	0.99
Selenium	1.00 (0.90-1.13)	0.94	0.99
Cobalt	0.93 (0.83-1.05)	0.23	0.71
Potassium	0.95 (0.85-1.07)	0.40	0.78
Uranium	1.00 (0.89-1.12)	0.98	0.99
Niobium	1.03 (0.92-1.16)	0.62	0.88
Cesium	0.94 (0.84-1.06)	0.33	0.78
Copper	1.04 (0.92-1.16)	0.55	0.88
Sodium	0.87 (0.75-1.01)	0.06	0.28
Thallium	1.03 (0.92-1.16)	0.59	0.88
Barium	1.10 (0.98-1.23)	0.12	0.50
Tantalum [a]	1.50 (0.98-2.30)	0.06	0.28
Zinc	1.01 (0.90-1.14)	0.82	0.99
Arsenic	1.02 (0.91-1.14)	0.79	0.99
Boron	0.97 (0.86-1.08)	0.57	0.88
Chromium	1.06 (0.94-1.19)	0.36	0.78
Lithium	0.93 (0.83-1.04)	0.22	0.71
Molybdenum	1.02 (0.91-1.15)	0.72	0.99
Calcium	1.00 (0.89-1.12)	0.99	0.99
Magnesium	1.06 (0.94-1.19)	0.34	0.78
Cadmium	1.08 (0.96-1.22)	0.18	0.68
Iron	0.95 (0.85-1.06)	0.36	0.78
Lead	1.05 (0.94-1.18)	0.38	0.78
Manganese	1.04 (0.92-1.16)	0.55	0.88
Rubidium	1.01 (0.90-1.13)	0.88	0.99

[a] For elements with low detection rates, effects estimates were calculated by comparing the detectable group to the undetectable group.

[b] Adjusted for age, sex, body mass index, cigarette smoking, alcohol drinking, creatinine clearance, C-reactive protein, hypertension, dyslipidemia, peripheral vascular disease, chronic obstructive pulmonary disease, glycated hemoglobin, fasting glucose, diabetes duration, and management of diabetes.

Supplementary Table 8

Incremental values of redox-related metal mixture and sORP beyond SYNTAX II score in predicting 2-year MACCE after PCI.

	SYNTAX II + redox-related metal mixture				SYNTAX II + sORP			
Events	< 20%	20%-50%	> 50%	Reclassified (%)	< 20%	20%-50%	> 50%	Reclassified (%)
**Individuals without MACCE, N**								
< 20%	396	58	0	13	399	55	0	12
20%-50%	125	302	37	35	98	327	39	30
> 50%	1	38	19	67	0	28	30	48
**Individuals with MACCE, N**								
< 20%	48	21	0	30	45	24	0	35
20%-50%	17	84	25	33	14	91	21	28
> 50%	0	16	25	39	0	13	28	32
**Effect size**	Estimate	Lower CI	Upper CI	*P* value	Estimate	Lower CI	Upper CI	*P* value
Δ C-statistic	0.033	0.007	0.059	0.012	0.026	0.004	0.047	0.018
IDI	0.026	0.012	0.04	< 0.001	0.016	0.007	0.026	0.001
NRI continuous	0.235	0.094	0.376	0.001	0.163	0.021	0.304	0.024
NRI categorical [a]	0.126	0.046	0.206	0.002	0.109	0.033	0.185	0.005

[a] The risk estimates of categorical NRI were defined as < 20%, 20-50%, and > 50% risk of respective outcomes.

Supplementary Table 9

Associations of redox-related metal mixture and sORP with cytokine concentrations

Variables	sORP			5-metal mixture		
	Percent change [a]	*P* value [a]	FDR	Percent change [a]	*P* value [a]	FDR
**JAK-STAT pathway**						
EGF	-0.3 (-8.3, 8.4)	0.95	0.98	6.8 (-7.9, 24.0)	0.38	0.86
IFN-α2	9.2 (-4.1, 24.5)	0.18	0.50	21.5 (-3.6, 53.1)	0.10	0.45
IFN-γ	-1.9 (-17.2, 16.2)	0.82	0.96	20.3 (-10.9, 62.4)	0.23	0.75
IL-2	3.9 (-13.0, 24.2)	0.67	0.96	-6.6 (-31.9, 28.1)	0.67	0.98
IL-4	-1.0 (-7.3, 5.8)	0.77	0.96	-6.2 (-16.5, 5.5)	0.29	0.75
IL-5	4.8 (-8.8, 20.4)	0.51	0.81	-1.2 (-22.8, 26.4)	0.92	0.98
IL-6	1.7 (-7.0, 11.1)	0.72	0.96	2.0 (-12.9, 19.5)	0.81	0.98
IL-10	-8.3 (-23.2, 9.4)	0.34	0.74	2.2 (-25.4, 39.9)	0.89	0.98
IL-12p40	-1.9 (-9.9, 6.9)	0.66	0.96	3.2 (-11.3, 20.2)	0.68	0.98
IL-12p70	-2.0 (-18.4, 17.7)	0.83	0.96	-2.1 (-29.3, 35.6)	0.90	0.98
IL-13	-1.2 (-11.6, 10.4)	0.83	0.96	-10.5 (-26.5, 9.0)	0.27	0.75
IL-15	28.2 (5.9, 55.0)	0.011	0.07	4.3 (-25.6, 46.3)	0.81	0.98
Pathway effect	0.4 (-2.8, 3.6)	0.81	0.96	-0.1 (-5.8, 5.7)	0.99	0.99
**NF-κB pathway**						
sCD40L	6.0 (0.5, 11.8)	0.005	**0.036**	8.2 (-1.5, 19.0)	0.10	0.45
IL-1β	21.2 (9.7, 33.8)	<0.001	**0.003**	22.5 (2.6, 46.1)	0.025	0.23
IL-8	18.8 (6.3, 32.8)	0.002	**0.018**	31.0 (7.5, 59.5)	0.007	0.08
MIP-1β	15.7 (0.4, 33.4)	0.044	0.18	31.5 (2.2, 69.3)	0.033	0.24
TNF-α	24.4 (10.8, 39.8)	<0.001	**0.003**	37.9 (12.1, 69.6)	0.002	**0.036**
Pathway effect	16.0 (7.5, 24.4)	<0.001	**0.001**	21.7 (8.9, 34.6)	0.001	**0.036**
**Chemokine pathway**						
IL-8	18.8 (6.3, 32.8)	0.002	**0.018**	31.0 (7.5, 59.5)	0.007	0.08
MCP-1	16.7 (-1.8, 38.7)	0.08	0.26	35.0 (-0.6, 83.4)	0.055	0.33
MIG	-8.7 (-22.8, 8.0)	0.29	0.70	17.6 (-12.7, 58.4)	0.28	0.75
MIP-1α	-5.7 (-21.2, 12.8)	0.044	0.18	31.5 (2.2, 69.3)	0.033	0.24
MIP-1β	15.7 (0.4, 33.4)	0.52	0.81	-8.0 (-33.1, 26.4)	0.61	0.98
Pathway effect	7.3 (-4.8, 19.4)	0.24	0.61	14.1 (-6.6, 34.8)	0.18	0.65
**Adipocytokine pathway**						
Adiponectin	-3.8 (-12.3, 5.5)	0.41	0.79	3.5 (-12.2, 22.1)	0.68	0.98
Lipocalin2	4.0 (-5.8, 14.8)	0.44	0.79	-4.1 (-19.6, 14.3)	0.64	0.98
Resistin	2.9 (-3.0, 9.2)	0.35	0.74	-1.3 (-11.2, 9.6)	0.80	0.98
Adipsin	2.8 (-4.5, 10.5)	0.46	0.79	9.5 (-3.7, 24.7)	0.17	0.65
PAI1	6.2 (0.3, 12.3)	0.037	0.17	-2.4 (-11.7, 7.9)	0.64	0.98
Pathway effect	3.1 (-0.2, 6.3)	0.07	0.25	0.2 (-5.4, 5.9)	0.93	0.98
**Cholesterol metabolism**						
ApoA1	4.3 (-1.0, 9.9)	0.12	0.36	-4.2 (-12.6, 5.1)	0.37	0.86
ApoA2	-9.9 (-17.7, -1.4)	0.023	0.12	-0.5 (-15.2, 16.8)	0.95	0.98
ApoB	-0.2 (-9.1, 9.4)	0.96	0.98	-3.3 (-17.9, 14.0)	0.69	0.98
ApoC2	4.2 (-5.8, 15.2)	0.43	0.79	-1.4 (-17.6, 17.8)	0.87	0.98
ApoC3	-0.1 (-5.4, 5.5)	0.97	0.98	2.2 (-7.2, 12.7)	0.65	0.98
ApoE	0.2 (-6.4, 7.3)	0.95	0.98	5.2 (-6.8, 18.7)	0.41	0.87
Pathway effect	-0.1 (-3.9, 3.8)	0.98	0.98	-0.5 (-5.5, 4.5)	0.85	0.98

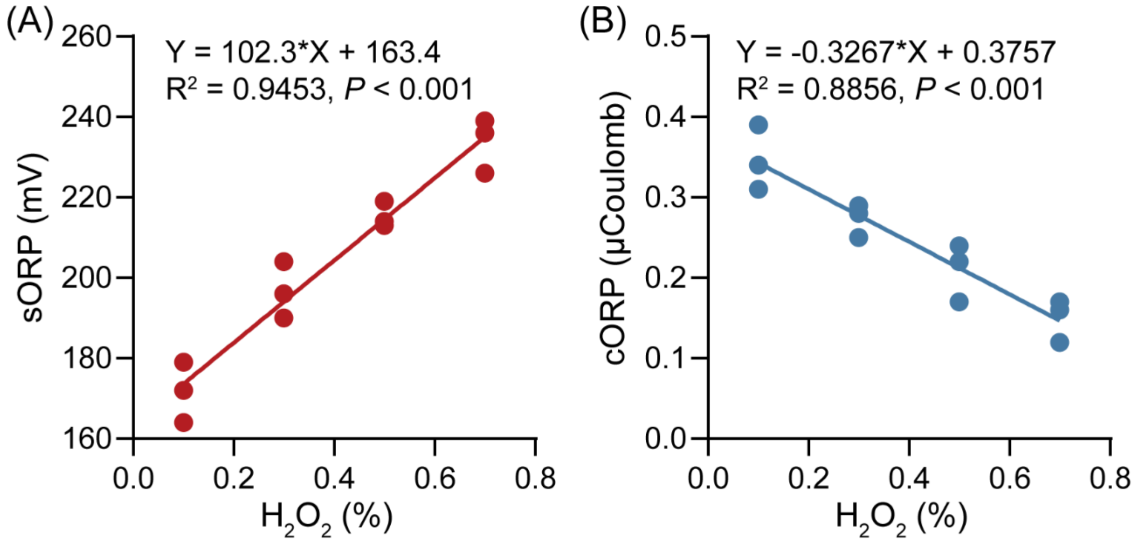

Supplementary Fig. 1

Effects of the addition of known oxidant H_2O_2 at incremental concentrations on static (**A**) and capacity (**B**) oxidation reduction potentials in a pooled human plasma sample.

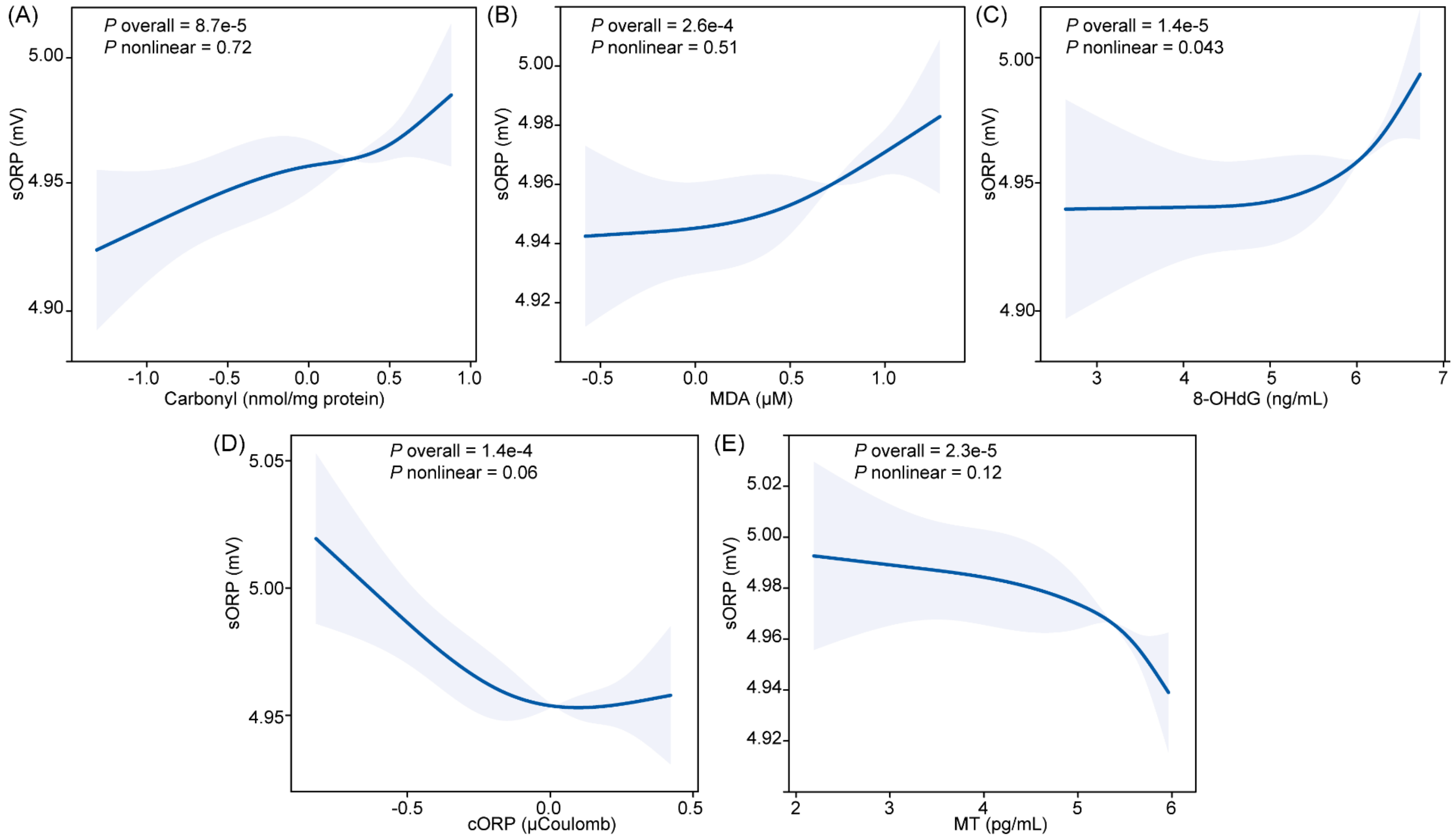

Supplementary Fig. 2

The dose-response associations of sORP with the oxidative stress markers protein carbonyl (A), MDA (B), and 8-OHdG (C) and the antioxidant indicators cORP (D) and MT (E).

All variables were ln-transformed before analysis. Models were adjusted for age, sex, body mass index, cigarette smoking, alcohol drinking, creatinine clearance, C-reactive protein, hypertension, dyslipidemia, peripheral vascular disease, chronic obstructive pulmonary disease, glycated hemoglobin, fasting glucose, diabetes duration, and management of diabetes.

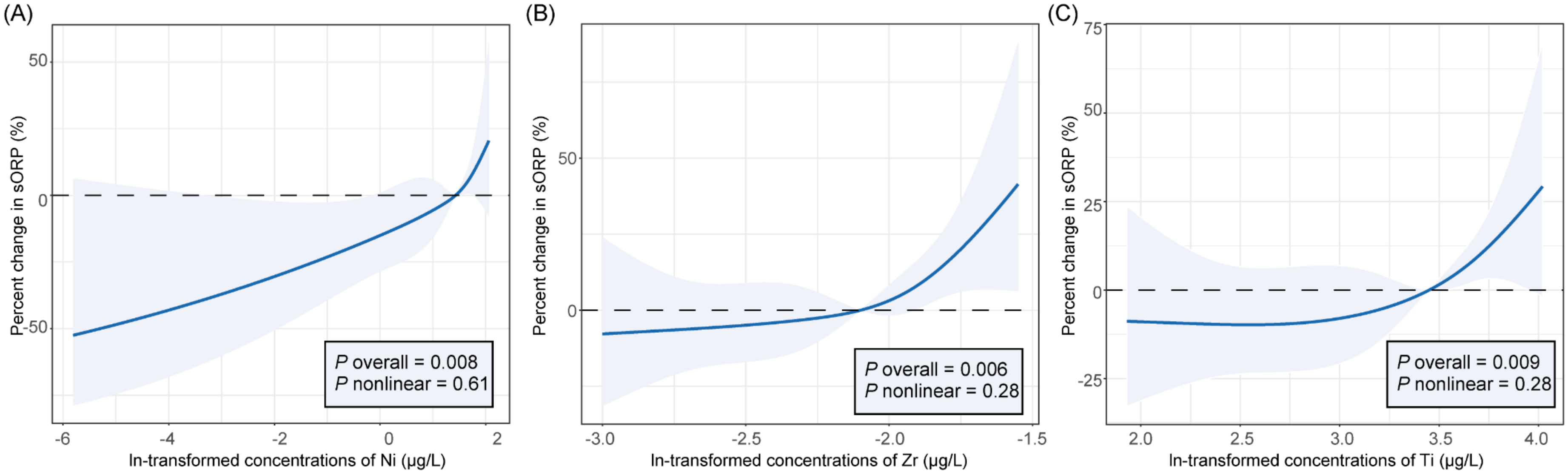

Supplementary Fig. 3

The restricted cubic spline assessing the dose-response associations of plasma nickel (A), zirconium (B), and titanium (C) with sORP values.

Models were adjusted for age, sex, body mass index, cigarette smoking, alcohol drinking, creatinine clearance, C-reactive protein, hypertension, dyslipidemia, peripheral vascular disease, chronic obstructive pulmonary disease, glycated hemoglobin, fasting glucose, diabetes duration, and management of diabetes. Percent changes in sORP values were computed using the formula: [exp (β) - 1] × 100%.

Characteristics	N	sORP values<br>Percent change (95%CI)	$P_{interaction}$
Overall	3142	20.5 (13.2, 28.2)	--
Age, years			
<60	1522	23.9 (13.5, 35.3)	0.34
≥60	1620	17.1 (7.2, 27.8)	
Sex			
Male	1782	14.5 (5.1, 24.7)	0.09
Female	1360	27.9 (16.9, 40.0)	
Cigarette smoking			
Never	1873	19.1 (10.2, 28.7)	0.64
Former	516	23.9 (6.7, 43.9)	
Current	753	21.7 (5.5, 40.4)	
Alcohol drinking			
Never	2151	18.4 (9.8, 27.7)	0.63
Former	146	47.4 (7.1, 102.8)	
Current	845	21.4 (8.0, 36.5)	
BMI, kg/m2			
<24	1338	19.9 (8.9, 31.9)	0.49
24-28	1385	15.0 (4.9, 26.0)	
>28	419	38.3 (13.6, 68.3)	
Hypertension			
No	1844	22.1 (12.7, 32.3)	0.59
Yes	1298	17.8 (6.8, 30.0)	
Dyslipidemia			
No	2355	21.0 (12.7, 29.9)	0.97
Yes	787	20.0 (5.4, 36.7)	
PAD			
No	2843	20.1 (12.5, 28.2)	0.76
Yes	299	22.3 (0.3, 49.1)	
T2DM duration, years			
<10	1566	27.7 (16.7, 39.7)	0.09
≥10	1576	14.5 (5.2, 24.7)	
T2DM Management			
Lifestyle	896	21.0 (8.0, 35.4)	0.67
Oral agent	1033	20.0 (8.0, 33.3)	
Insulin	777	16.3 (2.0, 32.7)	
Oral agent+Insulin	436	32.5 (10.5, 59.0)	
Glycemic control			
HbA1c<7%	1724	13.7 (-0.1, 29.4)	0.33
HbA1c≥7%	1418	22.2 (13.8, 31.1)	

0 45 90

Supplementary Fig. 4

Stratified analyses assessing the effect modification by baseline characteristics on the association between redox-related metal mixture and sORP.

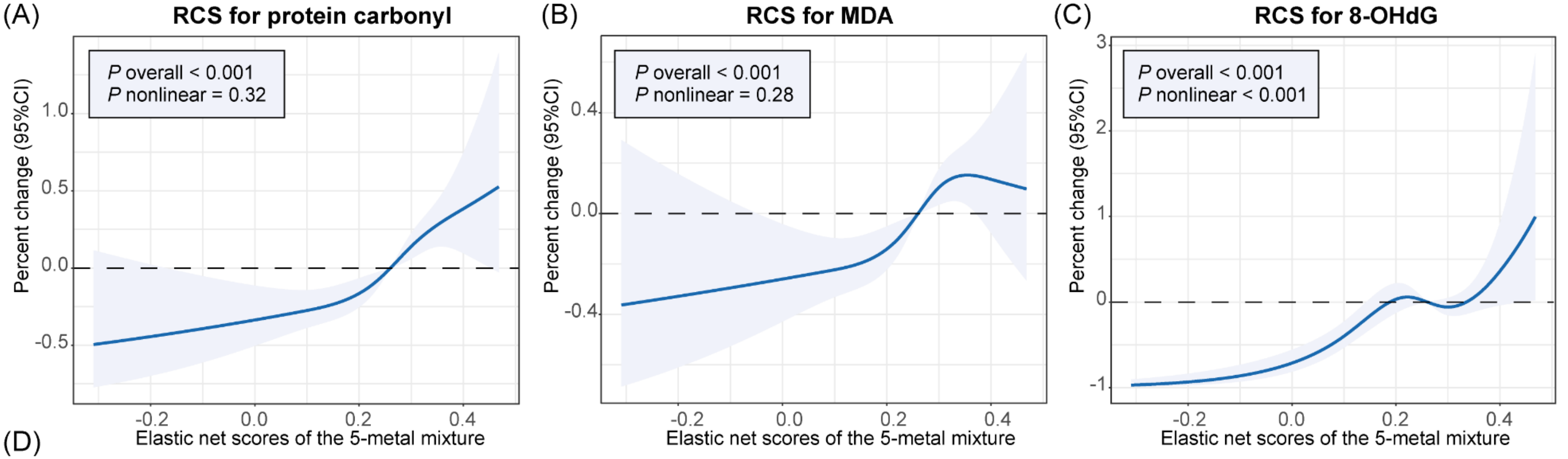

(D)

Quantile	Protein carbonyl: Percent change (95%CI)	Protein carbonyl: P_{trend} value	MDA: Percent change (95%CI)	MDA: P_{trend} value	8-OHdG: Percent change (95%CI)	8-OHdG: P_{trend} value
5th	-28 (-36, -18)	3.5e-08	-21 (-29, -12)	2.9e-05	-25 (-36, -12)	5.5e-04
15th	-15 (-20, -10)		-11 (-16, -6)		-13 (-20, -6)	
25th	Reference		Reference		Reference	
35th	17 (11, 25)		13 (6, 19)		15 (6, 25)	
45th	38 (23, 55)		27 (13, 42)		33 (13, 55)	
55th	62 (36, 94)		43 (21, 68)		53 (20, 94)	
65th	90 (50, 141)		61 (29, 100)		76 (28, 142)	
75th	124 (66, 201)		81 (37, 139)		102 (36, 201)	
85th	163 (84, 275)		104 (46, 184)		133 (44, 276)	
95th	209 (104, 367)		129 (55, 238)		168 (53, 369)	

(Forest plot axes: 0, 200, 400)

Supplementary Fig. 5

Joint effects of 5 redox-related metals on levels of protein carbonyl, MDA, and 8-OHdG by restricted cubic spline (A-C) and quantile g-computation (D).

Models were adjusted for age, sex, body mass index, cigarette smoking, alcohol drinking, creatinine clearance, C-reactive protein, hypertension, dyslipidemia, peripheral vascular disease, chronic obstructive pulmonary disease, glycated hemoglobin, fasting glucose, diabetes duration, and management of diabetes. Percent changes in sORP values were computed using the formula: [exp (β) - 1] × 100%.

Markers	Median (IQR)		Median Difference (95%CI)	*P* value
	No osbtructive CAD	Osbtructive CAD		
**Oxidative stress markers**				
sORP, mV	4.96 (4.80, 5.10)	5.01 (4.84, 5.16)	0.05 (0.04, 0.06)	<0.001
Carbonyl, nmol/mg protein	0.21 (-0.26, 0.54)	0.32 (-0.17, 0.65)	0.10 (0.07, 0.13)	<0.001
MDA, μM	0.69 (0.25, 1.01)	0.76 (0.33, 1.08)	0.07 (0.04, 0.10)	<0.001
8-OHdG, ng/mL	5.97 (5.32, 6.40)	6.10 (5.47, 6.47)	0.10 (0.06, 0.14)	<0.001
GSSG, μM	-3.08 (-3.24, -2.94)	-2.98 (-3.15, -2.85)	0.09 (0.07, 0.10)	<0.001
**Antioxidant markers**				
cORP, μCoulomb	0.05 (-0.22, 0.26)	-0.03 (-0.36, 0.22)	-0.08 (-0.10, -0.06)	<0.001
MT, pg/mL	5.47 (4.79, 5.84)	5.30 (4.66, 5.71)	-0.12 (-0.16, -0.08)	<0.001
GSH, μM	0.71 (0.49, 0.90)	0.65 (0.41, 0.86)	-0.06 (-0.08, -0.04)	<0.001

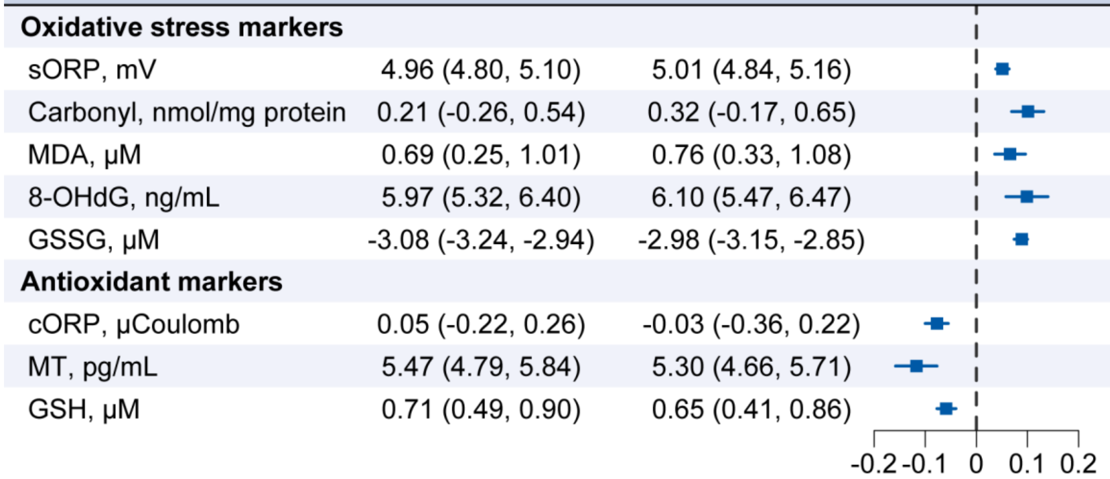

Supplementary Fig. 6

The median differences in concentrations of oxidative stress markers and antioxidant markers between type 2 diabetic patients with and without obstructive CAD.

All variables were ln-transformed before analysis. *P* values were estimated with the logistic regression model after adjusting for age, sex, body mass index, cigarette smoking, alcohol drinking, creatinine clearance, C-reactive protein, hypertension, dyslipidemia, peripheral vascular disease, chronic obstructive pulmonary disease, glycated hemoglobin, fasting glucose, diabetes duration, and management of diabetes.

Characteristics	N	Obstructive CAD OR (95%CI)	Obstructive CAD $P_{interaction}$	CAD index OR (95%CI)	CAD index $P_{interaction}$
Overall	3142	1.33 (1.25, 1.43)	--	1.22 (1.16, 1.29)	--
Age, years					
<60	1522	1.24 (1.13, 1.37)	0.06	1.18 (1.09, 1.28)	0.31
≥60	1620	1.43 (1.30, 1.57)		1.27 (1.17, 1.37)	
Sex					
Male	1782	1.35 (1.23, 1.47)	0.72	1.23 (1.14, 1.32)	0.73
Female	1360	1.32 (1.20, 1.46)		1.22 (1.12, 1.33)	
Cigarette smoking					
Never	1873	1.26 (1.16, 1.37)	0.07	1.17 (1.08, 1.25)	0.06
Former	516	1.47 (1.25, 1.73)		1.31 (1.14, 1.50)	
Current	753	1.42 (1.24, 1.64)		1.31 (1.17, 1.47)	
Alcohol drinking					
Never	2151	1.33 (1.22, 1.44)	0.74	1.20 (1.12, 1.28)	0.27
Former	146	1.49 (1.01, 2.20)		1.23 (0.91, 1.66)	
Current	845	1.37 (1.20, 1.56)		1.29 (1.16, 1.43)	
BMI, kg/m2					
<24	1338	1.32 (1.19, 1.46)	0.43	1.21 (1.11, 1.32)	0.67
24-28	1385	1.27 (1.14, 1.40)		1.19 (1.09, 1.30)	
>28	419	1.27 (1.01, 1.59)		1.15 (0.97, 1.36)	
Hypertension					
No	1844	1.32 (1.21, 1.44)	0.66	1.19 (1.10, 1.28)	0.17
Yes	1298	1.35 (1.22, 1.50)		1.27 (1.17, 1.39)	
Dyslipidemia					
No	2355	1.35 (1.25, 1.46)	0.47	1.23 (1.15, 1.31)	0.82
Yes	787	1.28 (1.12, 1.47)		1.21 (1.08, 1.36)	
PAD					
No	2843	1.34 (1.25, 1.44)	0.68	1.23 (1.16, 1.30)	0.89
Yes	299	1.26 (1.02, 1.57)		1.20 (1.00, 1.44)	
T2DM duration, years					
<10	1566	1.41 (1.28, 1.55)	0.13	1.27 (1.17, 1.37)	0.28
≥10	1576	1.27 (1.16, 1.40)		1.19 (1.10, 1.28)	
T2DM Management					
Lifestyle	896	1.44 (1.27, 1.64)	0.53	1.32 (1.19, 1.47)	0.45
Oral agent	1033	1.33 (1.18, 1.49)		1.22 (1.11, 1.35)	
Insulin	777	1.29 (1.12, 1.47)		1.18 (1.06, 1.33)	
Oral agent+Insulin	436	1.32 (1.11, 1.59)		1.14 (0.98, 1.33)	
Glycemic control					
HbA1c<7%	1724	1.37 (1.18, 1.58)	0.85	1.26 (1.11, 1.43)	0.94
HbA1c≥7%	1418	1.34 (1.24, 1.44)		1.23 (1.15, 1.31)	

Supplementary Fig. 7

Stratified analyses assessing the effect modification by baseline characteristics on the associations of redox-related metal mixture with obstructive CAD and CAD index.

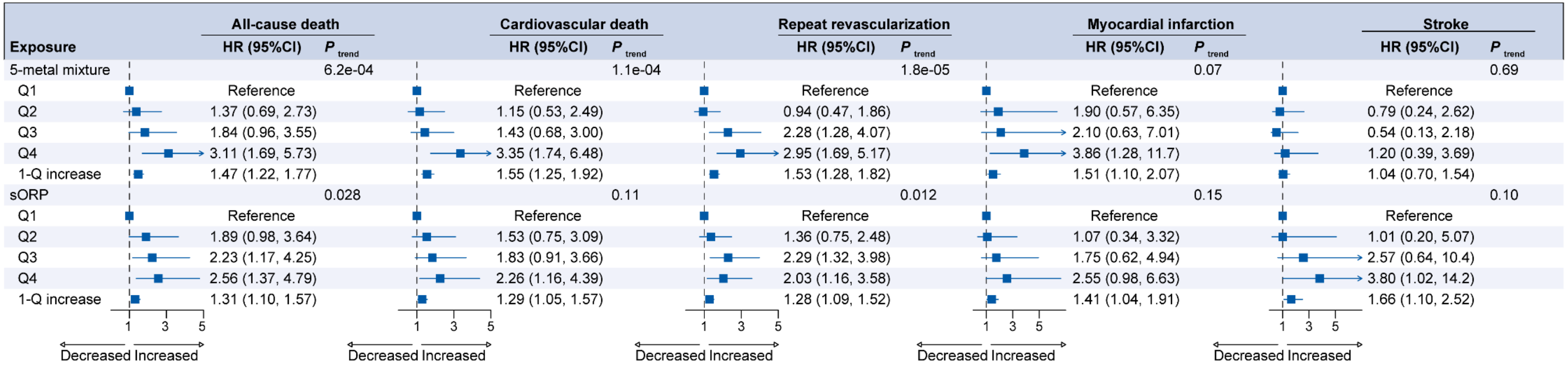

Exposure	All-cause death HR (95%CI)	All-cause death P_{trend}	Cardiovascular death HR (95%CI)	Cardiovascular death P_{trend}	Repeat revascularization HR (95%CI)	Repeat revascularization P_{trend}	Myocardial infarction HR (95%CI)	Myocardial infarction P_{trend}	Stroke HR (95%CI)	Stroke P_{trend}
5-metal mixture		6.2e-04		1.1e-04		1.8e-05		0.07		0.69
Q1	Reference		Reference		Reference		Reference		Reference	
Q2	1.37 (0.69, 2.73)		1.15 (0.53, 2.49)		0.94 (0.47, 1.86)		1.90 (0.57, 6.35)		0.79 (0.24, 2.62)	
Q3	1.84 (0.96, 3.55)		1.43 (0.68, 3.00)		2.28 (1.28, 4.07)		2.10 (0.63, 7.01)		0.54 (0.13, 2.18)	
Q4	3.11 (1.69, 5.73)		3.35 (1.74, 6.48)		2.95 (1.69, 5.17)		3.86 (1.28, 11.7)		1.20 (0.39, 3.69)	
1-Q increase	1.47 (1.22, 1.77)		1.55 (1.25, 1.92)		1.53 (1.28, 1.82)		1.51 (1.10, 2.07)		1.04 (0.70, 1.54)	
sORP		0.028		0.11		0.012		0.15		0.10
Q1	Reference		Reference		Reference		Reference		Reference	
Q2	1.89 (0.98, 3.64)		1.53 (0.75, 3.09)		1.36 (0.75, 2.48)		1.07 (0.34, 3.32)		1.01 (0.20, 5.07)	
Q3	2.23 (1.17, 4.25)		1.83 (0.91, 3.66)		2.29 (1.32, 3.98)		1.75 (0.62, 4.94)		2.57 (0.64, 10.4)	
Q4	2.56 (1.37, 4.79)		2.26 (1.16, 4.39)		2.03 (1.16, 3.58)		2.55 (0.98, 6.63)		3.80 (1.02, 14.2)	
1-Q increase	1.31 (1.10, 1.57)		1.29 (1.05, 1.57)		1.28 (1.09, 1.52)		1.41 (1.04, 1.91)		1.66 (1.10, 2.52)	

Supplementary Fig. 8

Prospective associations of redox-related metal mixture and sORP with individual components of MACCE after PCI.

Models were adjusted for age, sex, body mass index, cigarette smoking, alcohol drinking, creatinine clearance, C-reactive protein, hypertension, dyslipidemia, peripheral vascular disease, chronic obstructive pulmonary disease, glycated hemoglobin, fasting glucose, diabetes duration, and management of diabetes.

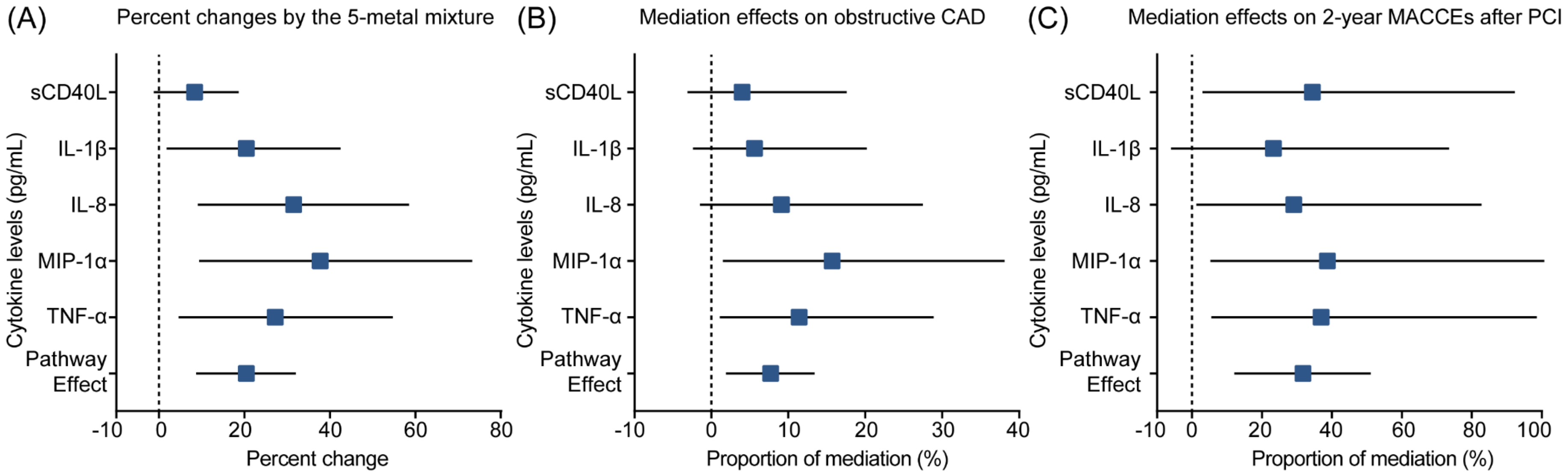

Supplementary Fig. 9

The mediation effects of cytokines within the NF-κB pathway after excluding the extreme outliers of cytokine measurements.

(**A**) Percent changes in cytokines within the NF-κB pathway by the redox-related metal mixture. (**B**) Mediation effects of cytokines within the NF-κB pathway on the cross-sectional association between the redox-related metal mixture and obstructive CAD. (**C**) Mediation effects of cytokines within the NF-κB pathway on the prospective association between the redox-related metal mixture and 2-year MACCE after PCI. A bootstrap method was used to estimate effect sizes and confidence intervals. Models were adjusted for age, sex, body mass index, cigarette smoking, alcohol drinking, creatinine clearance, C-reactive protein, hypertension, dyslipidemia, peripheral vascular disease, chronic obstructive pulmonary disease, glycated hemoglobin, fasting glucose, diabetes duration, and management of diabetes.

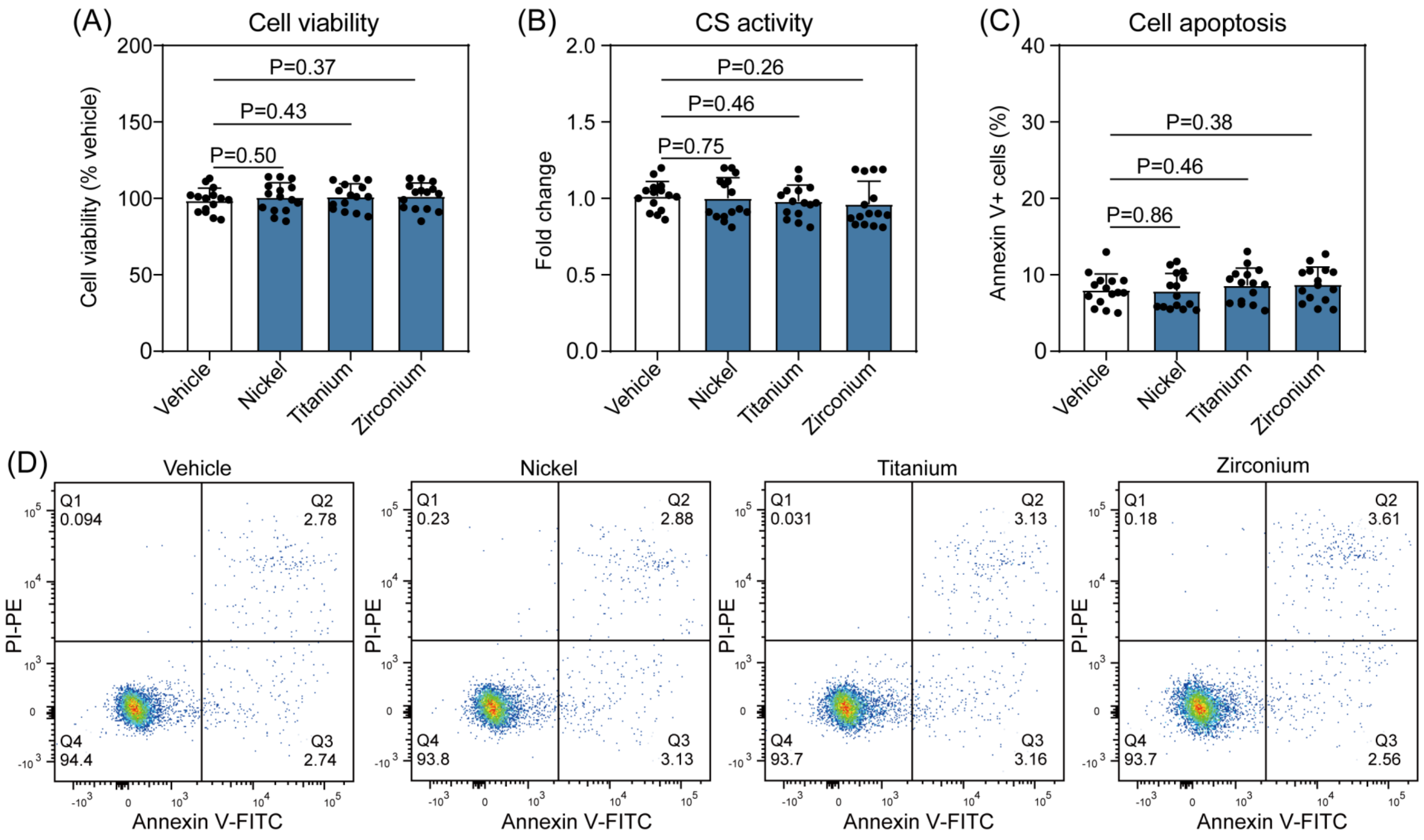

Supplementary Fig. 10

Exposures to redox related metals including nickel, titanium, and zirconium have no significant effects on cell viability (**A**), citrate synthase activity (**B**), and cell apoptosis (**C** and **D**) in PBMCs.

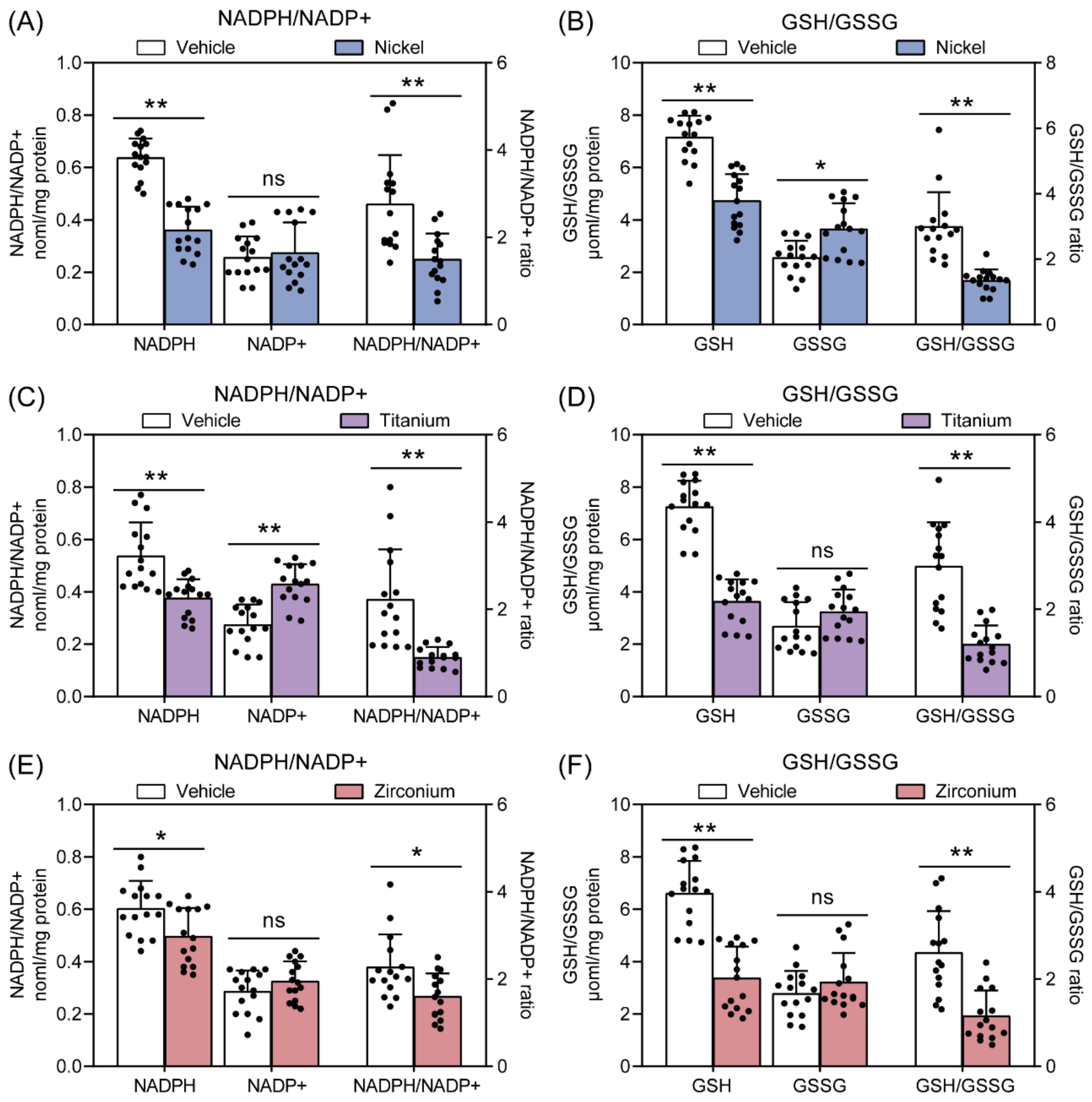

Supplementary Fig. 11

Exposures to redox related metals including nickel (**A** and **B**), titanium (**C** and **D**), and zirconium (**E** and **F**) have down-regulating effects on antioxidation systems of NADPH/NADP+ and GSH/GSSG in PBMCs. * $P < 0.05$; ** $P < 0.001$; ns, nonsignificant.

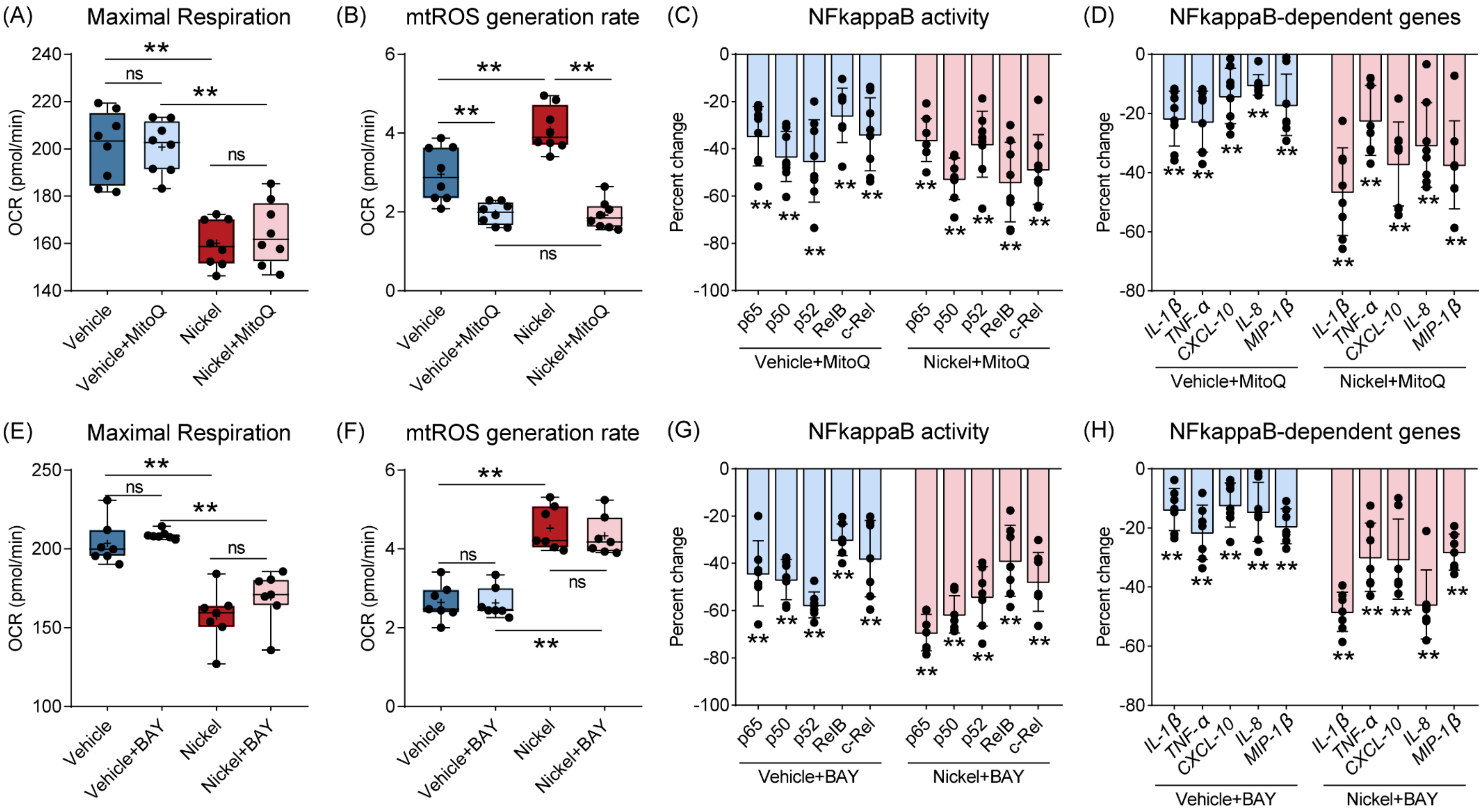

Supplementary Fig. 12

In nickel-exposed PBMCs, mtROS scavenging by MitoQ treatment fails to improve mitochondrial respiration (**A** and **B**), **but indeed inhibits NF-κB activity and NF-κB-dependent gene expression** (**C** and **D**), **while NF-κB inhibition by BAY 11-7082 has no significant effects on mitochondrial respiration and mtROS generation** (**E-H**). ** $P < 0.001$; ns, nonsignificant.

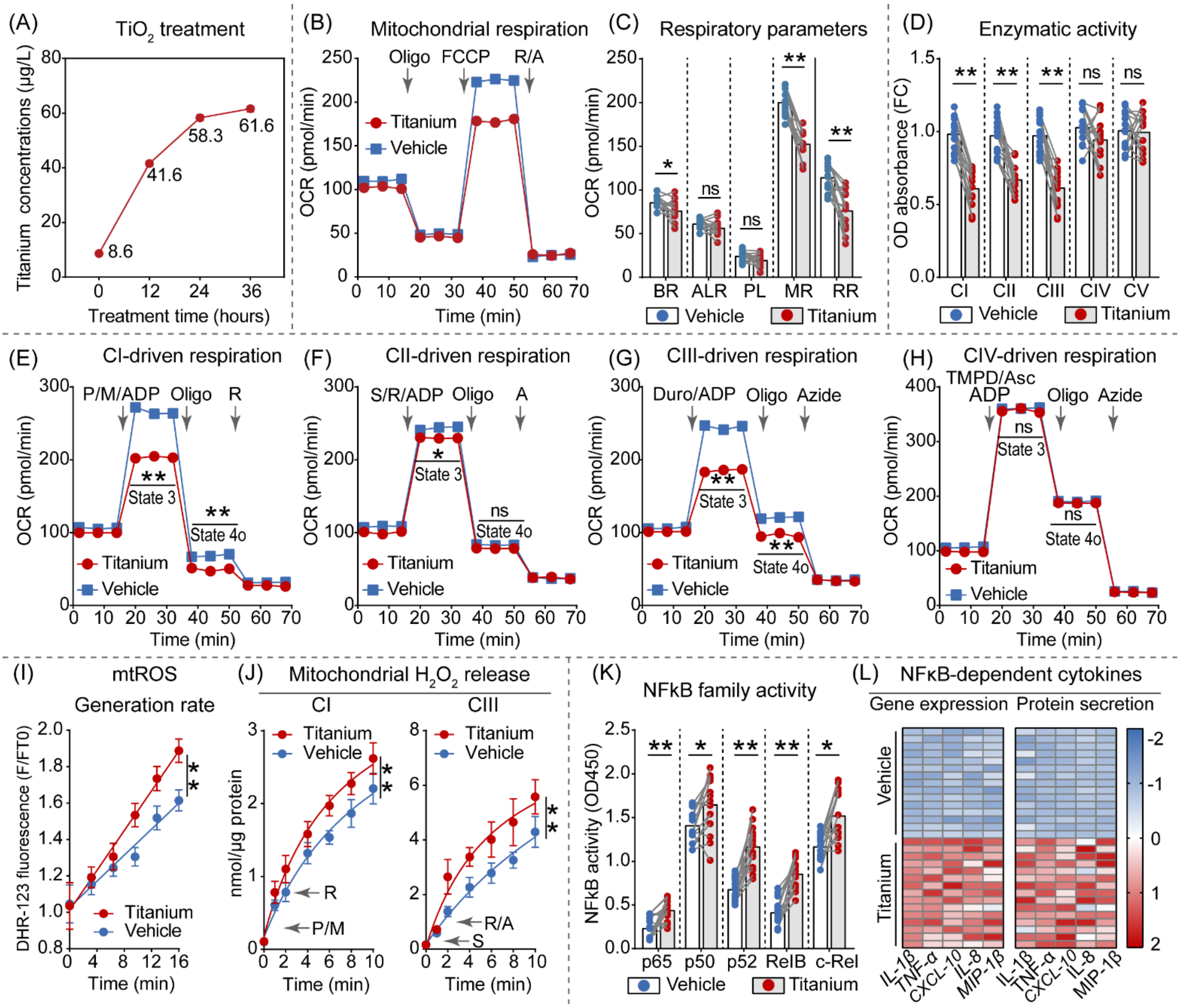

Supplementary Fig. 13

Exposure to titanium induces a similar pattern of bioenergetic dysfunction characterized by mitochondrial respiratory impairment, mtROS overproduction and NF-κB activation in PBMCs. (**A**) Time-dependent effects of TiO_2 treatment on concentrations of titanium in PBMCs. (**B** and **C**) Effects of titanium exposure on the overall capacity of mitochondrial respiration. (**D**) Effects of titanium exposure on the enzymatic activities of the ETC complexes (CI to CV). (**E-H**) Effects of titanium exposure on the respiratory activities of the ETC complexes (CI to CIV). (**I** and **J**) Effects of titanium exposure on mitochondrial superoxide generation and CI- and CIII-driven H_2O_2 release. (**K**) Effects of titanium exposure on the activities of the NF-κB family. (**L**) Effects of titanium exposure on mRNA expression and protein secretion of NF-κB-dependent cytokines. **N = 15 for each group.** * $P < 0.05$; ** $P < 0.001$; ns, nonsignificant.

Oligo indicates oligomycin; R, rotenone; A, Antimycin A; BR, basal respiration; ALR, ATP-linked respiration; PL, proton leak; MR, maximal respiration; RR, reserve respiration; P, pyruvate; M, malate; S, succinate; Duro, duroquinol; TMPD, N,N,N′,N′-tetramethyl-para-phenylene-diamine; Asc, ascorbate.

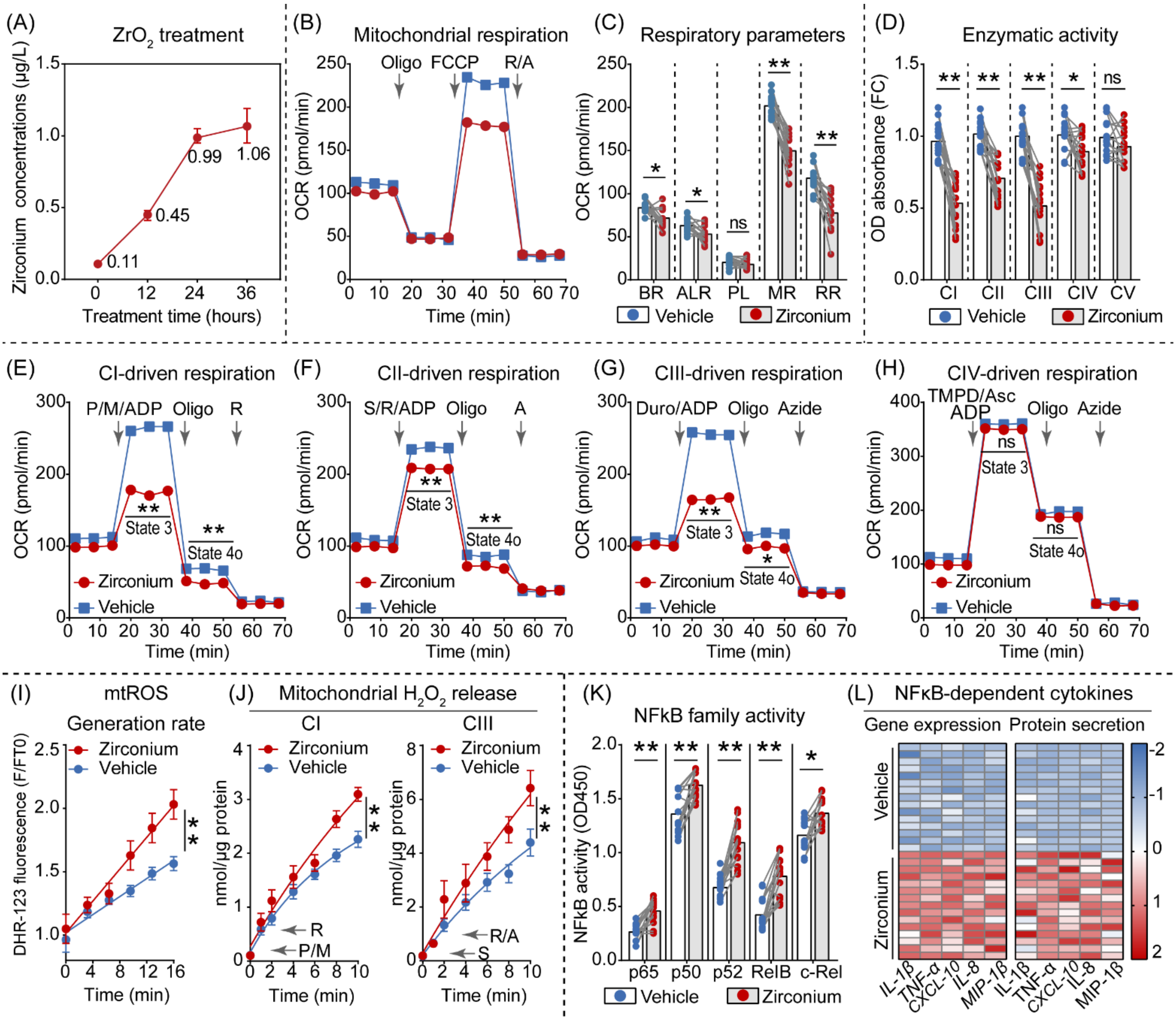

Supplementary Fig. 14

Exposure to zirconium induces a similar pattern of bioenergetic dysfunction characterized by mitochondrial respiratory impairment, mtROS overproduction and NF-κB activation in PBMCs. (**A**) Time-dependent effects of ZrO_2 treatment on concentrations of zirconium in PBMCs. (**B** and **C**) Effects of zirconium exposure on the overall capacity of mitochondrial respiration. (**D**) Effects of zirconium exposure on the enzymatic activities of the ETC complexes (CI to CV). (**E-H**) Effects of zirconium exposure on the respiratory activities of the ETC complexes (CI to CIV). (**I** and **J**) Effects of zirconium exposure on mitochondrial superoxide generation and CI- and CIII-driven H_2O_2 release. (**K**) Effects of zirconium exposure on the activities of the NF-κB family. (**L**) Effects of zirconium exposure on mRNA expression and protein secretion of NF-κB-dependent cytokines. **N = 15 for each group.** * $P < 0.05$; ** $P < 0.001$; ns, nonsignificant.

Oligo indicates oligomycin; R, rotenone; A, Antimycin A; BR, basal respiration; ALR, ATP-linked respiration; PL, proton leak; MR, maximal respiration; RR, reserve respiration; P, pyruvate; M, malate; S, succinate; Duro, duroquinol; TMPD, N,N,N′,N′-tetramethyl-para-phenylene-diamine; Asc, ascorbate.
